# SWEET1‐mediated glucose transport is crucial for energy availability in *Arabidopsis*

**DOI:** 10.1111/nph.70738

**Published:** 2025-11-18

**Authors:** Xueyi Xue, Jiankun Li, Ya‐Chi Yu, Li‐Qing Chen

**Affiliations:** ^1^ Department of Plant Biology University of Illinois at Urbana‐Champaign Urbana IL 61801 USA; ^2^ Sanya Institute China Agricultural University Sanya Hainan 572025 China

**Keywords:** abscisic acid, glucose, KIN10, seed germination, sugar transporter, SWEET1, TOR

## Abstract

- The stress‐responsive hormone abscisic acid (ABA) is known for its inhibitory effects on various physiological processes, including seed germination, often resulting in energy deprivation. Interestingly, ABA‐induced germination inhibition can be alleviated by exogenous glucose (Glc), mimicking a functional interplay between ABA and sugar signaling *in vivo*. However, it remains poorly understood which sugar transporter mediates Glc allocation under ABA treatment and how Glc counteracts the effects of ABA.
- To address this, we combined transcriptomic analyses, transport assays, physiological studies, and genetic approaches to identify and characterize processes involved in Glc allocation that counteract the inhibitory effects of ABA during seed germination in *Arabidopsis thaliana*.
- We identified the plasma membrane‐localized Glc uniporter SWEET1 as a key player in the Glc‐mediated suppression of ABA inhibition. The *sweet1* mutants showed delayed germination in the presence of ABA, reduced Glc transport, and slower root growth under energy starvation conditions. The suppression of ABA inhibited germination by Glc transported by SWEET1 appears to depend, at least partially, on Glc signaling through SnRK1 kinase pathway, including KIN10.
- Our findings demonstrate that SWEET1 is crucial for Glc suppression of ABA‐induced inhibition during seed germination and provide insight into sugar signaling pathways linked to environment‐ or ABA‐triggered energy deficiency.

## Abstract (zh)

- 逆境响应激素脱落酸(abscisic acid, ABA)以其对多种生理过程的抑制作用而著称，其中包括对种子萌发的抑制，常伴随能量匮乏的发生。值得注意的是，外源葡萄糖(Glc)可显著缓解ABA引起的萌发抑制，模拟了ABA与Glc在体内信号间的功能互作。然而，目前尚不清楚在ABA处理条件下哪种糖转运蛋白介导Glc的分配，以及Glc如何拮抗ABA作用的机制。
- 本研究通过结合转录组分析、转运活性测定、生理学实验及遗传学研究，系统解析了在拟南芥(*Arabidopsis thaliana*)种子萌发过程中，Glc分配是如何参与抵消ABA抑制效应的相关机制。
- 研究发现，质膜定位的Glc转运蛋白SWEET1是Glc介导缓解ABA抑制的关键因子。在有ABA存在的条件下，*sweet1*突变体表现出萌发延迟、Glc吸收能力下降以及能量饥饿条件下根系生长受限的表型。SWEET1介导的Glc转运对ABA抑制萌发的缓解作用，至少部分依赖于Glc‐SnRK1激酶通路(包括KIN10)介导的信号传导。
- 综上所述，本研究表明在种子萌发过程中，SWEET1介导的糖转运对ABA引起的抑制具有关键的缓解作用，并为理解由逆境或ABA激发的能量缺乏相关的糖信号通路提供了新的分子机制见解。

## Introduction

Seed germination is the first step of the plant life cycle for seed plants. It is a complex process of physiological and biochemical changes, including water uptake, metabolism reactivation, DNA repair, and mitochondria rebuilding, which leads to the emergence of the radicle from the seed (Bewley, 1997). Germination is strictly controlled by environmental stimuli and intrinsic hormones, such as abscisic acid (ABA) and gibberellin (GA) (Bewley, 1997; Tuan *et al*., 2018). Environmental stresses, such as drought, salinity, and high temperature, cause low water potential and cellular damage, restrict reserve remobilization, and inhibit seed germination (Cutler *et al*., 2010; Zhu, 2016). ABA, as a stress hormone, is induced by various environmental stresses, such as drought and salinity, and plays a vital role in various physiological processes, including maintaining seed dormancy and inhibiting seed germination (Finkelstein *et al*., 2002, 2008).

Angiosperm seeds generally consist of a seed coat, endosperm, and embryo, which are symplasmically isolated and, therefore, require membrane transporters to communicate nutrients. Our previous study showed that ABA similarly inhibited, but did not fully inhibit ^14^C‐labeled glucose (Glc) accumulation in both the embryo and endosperm/testa, likely by affecting Glc transporters present in both tissues (Xue *et al*., 2021). In addition, exogenous Glc was able to rescue ABA‐induced growth inhibition in both isolated embryos and whole seeds, indicating that the effect of ABA on Glc import in embryos is representative of its overall effect on the seed (Xue *et al*., 2021). Moreover, the endosperm plays a critical role in nutrient provision and hormone regulation of embryo development (Lee *et al*., 2010; Kang *et al*., 2015; Sanchez‐Montesino *et al*., 2019). However, it remains unclear which transporter is still functional to mediate Glc import into an embryo, thereby overcoming ABA‐induced inhibition.

ABA treatment can mimic abiotic stress conditions without causing visible cellular damage, making it a useful tool for studying stress‐related, ABA‐dependent responses. Previous studies have shown that low concentrations of Glc, sucrose (Suc), or fructose (Fru) can alleviate ABA‐induced inhibition of seed germination (Garciarrubio *et al*., 1997; Finkelstein & Lynch, 2000; Xue *et al*., 2021). This suggests that inhibition of germination by ABA reflects energy and nutrient limitations rather than a simple nutrient deficiency. In germinating seeds, higher levels of soluble sugars mobilized from internal reserves are positively associated with faster germination rates (Zhao *et al*., 2018). In starch‐rich seeds, these sugars mainly derive from starch degradation. However, mature Arabidopsis seeds contain minimal starch – restricted to the lower hypocotyl – and instead store lipids as their main carbon source (Streb & Zeeman, 2012; Chen *et al*., 2015). During germination, lipids are broken down into acetyl‐CoA, which feeds into the glyoxylate cycle to produce four‐carbon intermediates. These are converted to sucrose via gluconeogenesis (Quettier & Eastmond, 2009), then transported or further metabolized into Glc and Fru before being transported to support growth.

Several sugar signaling pathways in Arabidopsis have been reported, including Hexokinase 1 (HXK1) (Jang *et al*., 1997; Moore *et al*., 2003), the regulator of G‐protein signaling 1 (RGS1) (Chen *et al*., 2003), target of rapamycin (TOR) (Menand *et al*., 2002; Xiong & Sheen, 2012), and SNF1‐related protein kinase (SnRK1)‐mediated pathways (Le Guen *et al*., 1992; Baena‐Gonzalez *et al*., 2007). In yeast, both energy deprivation caused by Glc limitation and environmental stress, such as sodium ion stress and alkaline pH can activate the SNF1 signaling pathway (Woods *et al*., 1994; Hong & Carlson, 2007). The plant orthologs, the SnRK1s, exert similar responses to energy deprivation, such as hypoxia and extended darkness (Baena‐Gonzalez *et al*., 2007). SnRK1s and TOR are evolutionarily conserved protein kinases that sense energy status but play antagonistic roles in energy homeostasis through the control of gene expression (Baena‐Gonzalez *et al*., 2007; Xiong *et al*., 2013). It remains unclear which sugar signaling pathway is involved in the inhibitory effect of Glc on ABA during seed germination.

Our previous study showed that in ABA‐treated seeds, genes encoding sugar transport proteins 1 and 4 (STP1 and STP4) and SWEET15 were downregulated by ABA, which implies that they may contribute to reduced Glc absorption into ABA‐treated seeds and are unlikely to play a key role in mediating Glc transport for the antagonistic role of ABA (Xue *et al*., 2021). In this study, we report that *sweet1* mutants fail to restore germination under ABA treatment in the presence of Glc. We also demonstrate that SWEET1 transports Glc and is localized to the plasma membrane of embryo epidermal and cortical cells in germinating seeds. This suggests SWEET1 is primarily responsible for Glc transport into the embryo, thereby providing the energy needed to overcome ABA inhibition during germination. Our transcriptomic data show that the SnRK1 pathway was differentially regulated by Glc + ABA in WT and in *sweet1*. The genetic analysis reveals that mutation of *SNF1 kinase homolog 10* (*KIN10*), encoding a key catalytic subunit of the SnRK1 complex, compromises the Glc effects on antagonizing ABA inhibition.

## Materials and Methods

### Plant materials and growth conditions

*Arabidopsis thaliana* ecotype Col‐0 was used as wild‐type (WT). All plants used in this study were in the Col‐0 background. The *sweet1* (SALK_029479), *stp1‐1* (SALK_048848), *stp13‐1* (SALK_045494), *plt4* (SAIL_759_E05), *plt5* (SALK_050162), *abi5‐8* (SALK_013163), and *tor‐es* were obtained from the Arabidopsis Biological Resource Center (ABRC). The *kin10* (GABI_579_E09) and *p35S:KIN10‐myc* were obtained from Dr Ming‐Yi Bai. The T‐DNA insertion site of *sweet1* was determined by amplifying the flanking region and sequencing the PCR products. Seeds were surface sterilized by 70% ethanol for 5 min, rinsed with distilled water three times, and then kept at 4°C for 3 d for imbibition. The seeds were then sown on ½‐strength Murashige & Skoog (½MS) medium with 1% agar. All stocks added to sterilized media (sugars, ABA, and estradiol) were sterilized by a 0.22‐μm filter. Plates were incubated in the growth chamber with constant light (100 μmol m^−2^ s^−1^) at 22°C.

### Plasmid constructs and plant transformation

The 4056‐bp genomic fragment containing the *SWEET1* promoter and coding region was amplified using primers SWEET1CMF‐1 and SWEET1CMR‐1 and cloned into the entry vector pDORN221‐f1. The *pSWEET1:gSWEET1* cassette was switched to the binary vectors pEG‐TW1‐EYFP and pMDC163 via LR reaction to make *pSWEET1:gSWEET1‐YFP* and *pSWEET1:gSWEET1‐GUS*, respectively. The fragment *pSWEET1:gSWEET4* was synthesized by replacing the *SWEET1* exonic sequences with the *SWEET4* exonic sequences and retaining the *SWEET1* introns and cloned into the entry vector pDORN221‐f1 to obtain *pDONR221‐f1/pSWEET1:gSWEET4*. The *pSWEET1:gSWEET4* cassette was switched to the pEG‐TW1‐EYFP vector by LR reaction. The *sweet1_crispr* lines were generated via the pCAMBIA1300/*pYAO*:hSpCas9 system (Yan *et al*., 2015) using primers SWT1_CRISPR‐F and SWT1_CRISPR‐R. Arabidopsis plants were transformed using standard floral dip methods. Transformants were selected on ½MS supplemented with 25 μg ml^−1^ glufosinate ammonium or 50 μg ml^−1^ hygromycin. See Supporting Information Dataset S1 for oligonucleotide sequences.

### Germination assays

After 3‐d imbibition at 4°C in the dark, at least 50 seeds for each genotype were sown on ½MS medium supplemented with or without ABA, sugar, or other chemicals. Germination is defined as the first sign of radicle tip emergence from the endosperm, which is counted every 12 h. The germination rate was calculated based on at least three replicates. Without being specified in this text, either 60 mM Glc, 2 μM ABA, or 2 μM ABA plus 60 mM Glc (ABA + Glc) was applied for this study. Seeds in each germination assay were harvested from the same batch of plants, which were grown in the same room and under consistent controlled growth conditions. We observed that different batches and storage periods of ABA may slightly affect the suppression of germination.

### Glc uptake assays

Seeds were sown on ½MS medium with 1% agar and grown under constant light at 22°C for 24 h in the chamber, then transferred to fresh liquid ½MS medium containing 12.8 μM D‐[^14^C]‐Glc (NEC‐042X; PerkinElmer, Waltham, MA, USA) with or without 5 μM ABA, or in the presence of 60 mM cold Glc, for 6 h with 200 rpm shaking. Seeds were rinsed three times and ground with a pestle in a 1.5‐ml tube. The lysate solution was transferred into a 5‐ml scintillation vial, followed by the addition of 200 μl 1% bleach. Radioactivity was measured by liquid scintillation spectrometry (LS 6500 multi‐purpose scintillation counter; Beckman Coulter, Brea, CA, USA) for 3 min per vial. For the uptake assay in germinating seeds, seeds were sown on ½MS medium with 1% agar supplemented with D‐[^14^C]‐Glc. Ten newly sprouted seedlings from each genotype per replicate were collected at 60 h after imbibition (HAI).

### GUS histochemistry

*pSWEET1:gSWEET1‐GUS* seeds were surface‐sterilized and dissected by a needle to separate the embryos and seed coats (endosperm + testa) at the indicated time points. The isolated embryos and seed coats were submerged in 0.5 mg ml^−1^ X‐Gluc solution (0.1 M sodium phosphate buffer, pH 7.0, 10 mM EDTA, 0.1% Triton X‐100, 0.5 mM potassium ferrocyanide, 0.5 mM potassium ferricyanide), subjected to vacuum infiltration a few times, and kept at 37°C for 12 h. After that, the materials were destained in 70% ethanol.

### Confocal imaging

After imbibition, *pSWEET1:gSWEET1‐YFP*/*sweet1* seeds were sown on ½MS medium with or without treatments. The embryos were isolated at the indicated time points. The fluorescence in the embryonic tissues was observed and imaged using a Carl Zeiss LSM710 confocal microscope. Fluorescent proteins were excited at 514 nm, and emissions were collected at 540 nm. The confocal images were processed using ImageJ (Fiji).

### Yeast complementation assay

The CDS fragments of *SWEET1* and *SWEET4* were cloned into the pDONR221‐f1 vector and then switched to the pDRf1‐GW vector via LR reaction. These constructs were transferred into *Saccharomyces cerevisiae* strain EBY.VW4000 using the LiAc‐mediated method. The positive control pDRf1‐GW/HXT5 and the yeast growth assay on plates supplemented with 2% different sugars were described in a previous study (Chen *et al*., 2010).

### Transcriptomic analysis

After 3‐d imbibition at 4°C, WT and *sweet1* seeds were sown on ½ MS medium and incubated for 24 h in a growth chamber with constant light at 22°C. Seeds were collected and transferred to liquid ½MS medium under treatment of either 60 mM Glc, 5 μM ABA or 60 mM Glc plus 5 μM ABA for 6 h. RNA was extracted by RNeasy PowerPlant Kit (13500‐50; Qiagen) according to the manufacturer's instructions. DNA was removed by Turbo DNA‐free kit (AM1907; Invitrogen). A total of 1 μg total RNA was used for library preparation. The libraries were constructed using KAPA mRNA Hyper Prep kit (KK8581; Roche) with KAPA Dual‐indexed Adapter kit for Illumina platforms (KK8722; Roche). Libraries were pooled at equimolar ratios and sequenced on an Illumina NovaSeq 6000 platform with paired‐end reads at the UIUC Roy J. Carver Biotechnology Center. Gene expression levels were quantified using Kallisto v 0.44.033 (Bray *et al*., 2016) by mapping reads to *Arabidopsis thaliana* primary transcript sequences (v11). The differentially expressed genes (DEGs) were screened out using the Sleuth program (Pimentel *et al*., 2017) with > 10 counts per million reads in at least 50% samples, a |*b*| ≥ 0.2, and a *q*‐value < 0.05. Samples were evaluated based on pair‐wise comparison between different treatments, and outliers within each treatment condition were removed before further principal component analysis. Hierarchical clustering was performed using the gplots package in R based on the TPM data. GO analysis was performed using PANTHER35 (*P* < 0.05). The transcriptomic data from Col‐0 under different treatments were published in Xue *et al*. (2021), although the *sweet1* dataset was collected together.

### Total protein extraction and immunoblotting

WT and *sweet1* seeds were sown on ½MS medium supplemented with Glc, ABA, or ABA + Glc, and samples were collected at 60 HAI. Total proteins were extracted in a buffer containing 50 mM Tris–HCl (pH 7.5), 150 mM NaCl, 10 mM MgCl_2_, 0.1% Tween 20, 1 mM PMSF, and 1% protease inhibitor cocktail. After centrifugation at 14 000 ***g*** for 15 min at 4°C, supernatants were transferred to new tubes. Equal amounts of total protein were loaded and separated by electrophoresis on a 12% SDS‐PAGE gel. ABI5 levels were determined by immunoblotting with the primary antibody against ABI5 (ab98831; Abcam, Cambridge, UK).

## Results

### SWEET1 is involved in antagonizing ABA‐inhibited seed germination

**Fig. 1:**
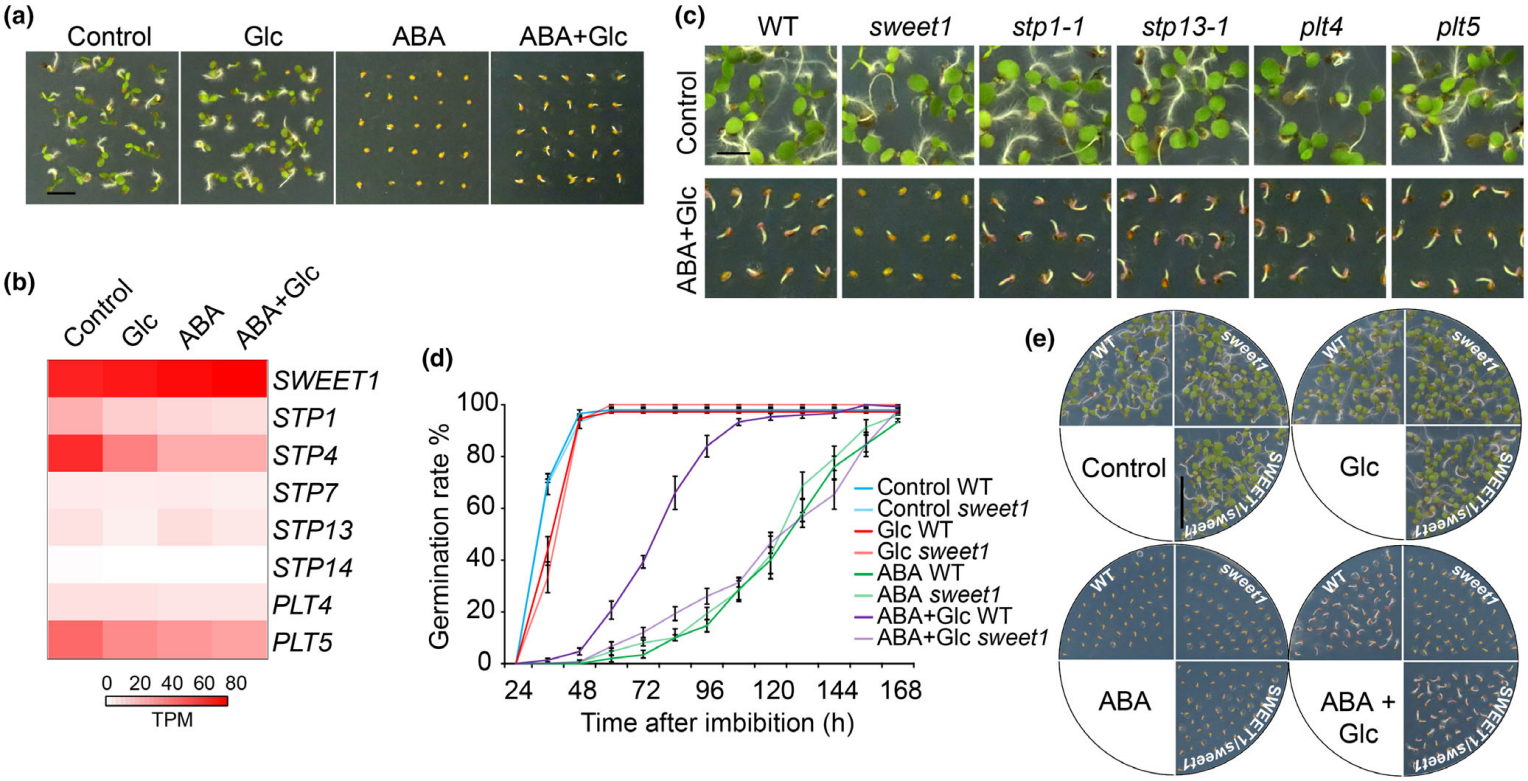
SWEET1 contributes to glucose (Glc) suppression of abscisic acid (ABA) inhibition on seed germination. (a) Glc antagonizes ABA inhibition on germination in *Arabidopsis thaliana*. After a 3‐d imbibition, wild‐type (WT) (Col‐0) seeds were placed on ½‐strength Murashige & Skoog (½MS) medium supplemented with either Glc or ABA or both. Images were captured at 4 d after imbibition (DAI). Bar, 5 mm. (b) Transcript levels of Glc transporter genes upon treatments in germinating seeds at 30 h after imbibition. Values are transcripts per million (TPM). (c) Phenotypes of sugar transporter mutants upon ABA + Glc treatment. After 3‐d imbibition, mutant seeds were sown on ½MS medium with either Glc, ABA, or ABA + Glc. Mutants showed comparable phenotypes to WT under all conditions except *sweet1* under ABA + Glc condition. Images were captured on 7 DAI. Bar, 5 mm. (d) Germination rate of WT and *sweet1* under treatments. Glc fails to promote germination of *sweet1* in the presence of ABA. Values are means ± SE (*n* = 3). (e) Phenotypes of *sweet1* upon treatments for 7 d. Seeds of WT, *sweet1,* and *pSWEET1:gSWEET1‐YFP/sweet1* were grown on the indicated medium. SWEET1‐YFP can rescue *sweet1* defects on the ABA + Glc medium. Bar, 1 cm.

After a 3‐d imbibition, WT (Col‐0) seeds were sown on ½MS medium supplemented with either Glc, ABA, or ABA + Glc. Germination inhibition by ABA was relieved by Glc supplementation (Fig. 1a). Using a noninvasive FRET Glc sensor that can monitor steady cytosolic Glc levels, we previously showed that Glc breaks ABA inhibition on seed germination by overcoming the local restrictions on Glc availability (Xue *et al*., 2021). This phenomenon suggests that at least one Glc transporter still functions under ABA treatment. To investigate which sugar transporter mediates Glc uptake into seeds to overcome ABA inhibition during germination, we checked the expression patterns of known Glc transporter gene families from our transcriptome dataset that were collected from WT seeds treated for 6 h at 30 hours after imbibition (HAI) (Xue *et al*., 2021). Under the control condition, mRNA transcripts of *SWEET1*, *sugar transport protein 1* (*STP1*), *STP4*, *STP7*, *STP13*, *STP14*, *polyol transporter 4* (*PLT4*), and *PLT5* were detected before germination (Fig. S1a–c). Among them, *STP1*, *STP4*, *PLT4*, and *PLT5* were downregulated to a similar degree by ABA and ABA + Glc treatments in germinating seeds (Fig. 1b) (Xue *et al*., 2021), which suggests that they are unlikely to be involved in Glc suppression of ABA inhibition. Only the *SWEET1* transcript level remained high upon ABA treatment with or without Glc, which indicates that SWEET1 may maintain its full function under all conditions (Fig. 1b). Moreover, *SWEET1* was upregulated, while *STP1*, *STP4*, and *STP14* were downregulated by ABA + Glc in the seedling stage (Fig. S1d) (Li *et al*., 2006). To validate their functions, we tested the germination phenotypes of T‐DNA insertional mutants of *SWEET1*, *STP1*, *STP13*, *PLT4*, and *PLT5* under these conditions. Only the *sweet1* mutant failed to relieve ABA inhibition on germination by Glc. Other mutants we tested did not show any visible differences compared to WT under treatment (Fig. 1c). The *sweet1* (SALK_029479) is a null mutant since no transcript was detected (Fig. S2a,b). Comparison of the germination rate over time between *sweet1* and WT upon the different treatments clearly showed that germination of both WT and *sweet1* was suppressed by ABA up to 7 d after imbibition (DAI), and that Glc rescued germination of the WT but not *sweet1* under ABA treatment (Fig. 1d). This partial rescue of WT and lack of rescue for *sweet1* by Glc was further confirmed by a second mutant generated by CRISPR/Cas9 editing (Fig. S2c), consistent with what was shown in Fig. 1(c). As we expected, late germination and retarded seedling growth of *sweet1* on media containing ABA + Glc could be fully rescued by SWEET1‐YFP translational fusion under the control of the native promoter (*pSWEET1:gSWEET1‐YFP*) (Fig. 1e). Therefore, we concluded that SWEET1 contributes to Glc allocation in ABA‐treated seeds.

### The *sweet1* mutant impairs Glc uptake and allocation

**Fig. 2:**
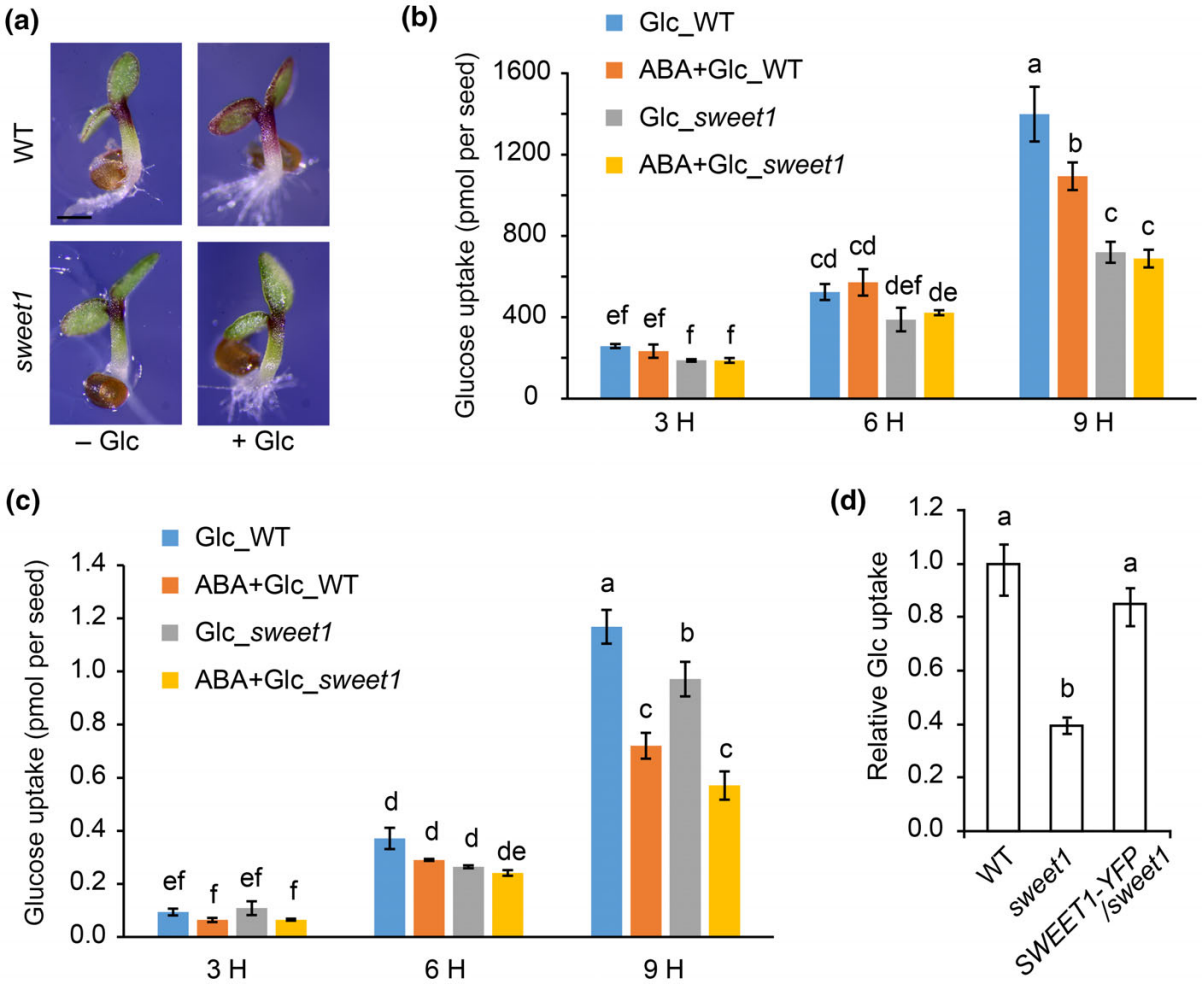
Glucose (Glc) uptake is impaired in the *sweet1* mutant. (a) Phenotypes of wild‐type (WT) and *sweet1* on medium supplied with 60 mM Glc. More anthocyanin accumulates in the hypocotyl region of 3‐d‐old WT seedlings. Bar, 0.5 mm. (b, c) Glc uptake assay in WT and *sweet1* upon abscisic acid (ABA) treatment with (b) or without (c) 60 mM cold Glc in the medium. Arabidopsis seeds at 24 h after imbibition were collected and transferred to a liquid ½‐strength Murashige & Skoog medium with incubation for 3, 6, and 9 h. Values are means ± SE (*n* = 3). Different lowercase letters indicate significantly different means at *P* < 0.05 by one‐way ANOVA Tukey test. (d) Glc uptake assay of WT, *sweet1,* and *SWEET1‐YFP/sweet1*. Relative levels of Glc uptake in *sweet1* and *SWEET1‐YFP/sweet1* were normalized to the value of WT. Values are means ± SE (*n* = 4). Different lowercase letters indicate significantly different means at *P* < 0.05 by one‐way ANOVA Tukey test.

In addition to germination phenotypes mentioned above, we observed that the hypocotyl region of 3‐d‐old SWEET1 seedlings accumulated less anthocyanin than WT, likely due to impaired Glc uptake (Fig. 2a), aligning with previous findings that a high concentration of Glc can trigger anthocyanin accumulation in plants (Solfanelli *et al*., 2006; Hu *et al*., 2016). This result demonstrates that SWEET1 plays a role at the post‐germination growth stage.

Since SWEET1 transports Glc (Chen *et al*., 2010) and functions during and after seed germination, we directly evaluated how much SWEET1 contributes to overall Glc uptake in germinating seeds. The uptake of radioactive Glc was measured in WT and *sweet1* upon ABA treatment with (Fig. 2b) or without (Fig. 2c) 60 mM cold Glc. Under the 60 mM Glc condition, the amount of Glc absorbed by the *sweet1* mutant was only half that of WT after incubation for 9 h (Fig. 2b). ABA significantly suppressed Glc uptake in both WT and *sweet1*, although the difference is not as great for the mutant with and without ABA. These data indicate that SWEET1 is the main contributor to Glc uptake in the presence of 60 mM Glc (Fig. 2b). Under the limited Glc condition with radioactively labeled Glc only (*c*. 12.8 μM Glc in the final reaction buffer), the *sweet1* took up 85% of the Glc absorbed by WT (Fig. 2c), which is consistent with the presence of other high‐affinity Glc transporters, such as the STPs (Buttner, 2010). Under the low Glc concentration, the inhibition of Glc uptake by ABA in both WT and *sweet1* is likely due to ABA‐mediated downregulation of *STP* expression (Xue *et al*., 2021). Our data suggest that SWEET1 functions better when Glc is in the millimolar range, which aligns with its affinity for Glc at *c*. 9 mM (Chen *et al*., 2010). By contrast, STPs generally function at a low Glc concentration, compatible with their high affinity with *K*_m_ values in the micromolar range (Buttner, 2010). To test the Glc uptake during germination, seeds were sown on ½MS medium supplemented with D‐[^14^C]‐Glc, and seeds that had just germinated were collected at 60 HAI. The Glc uptake defect of the *sweet1* mutant under ABA + Glc was fully complemented by *pSWEET1:gSWEET1‐YFP* (Fig. 2d). This complementation further supports the idea that SWEET1 plays a critical role in countering ABA inhibition.

### Cell‐type expression of *SWEET1*

**Fig. 3:**
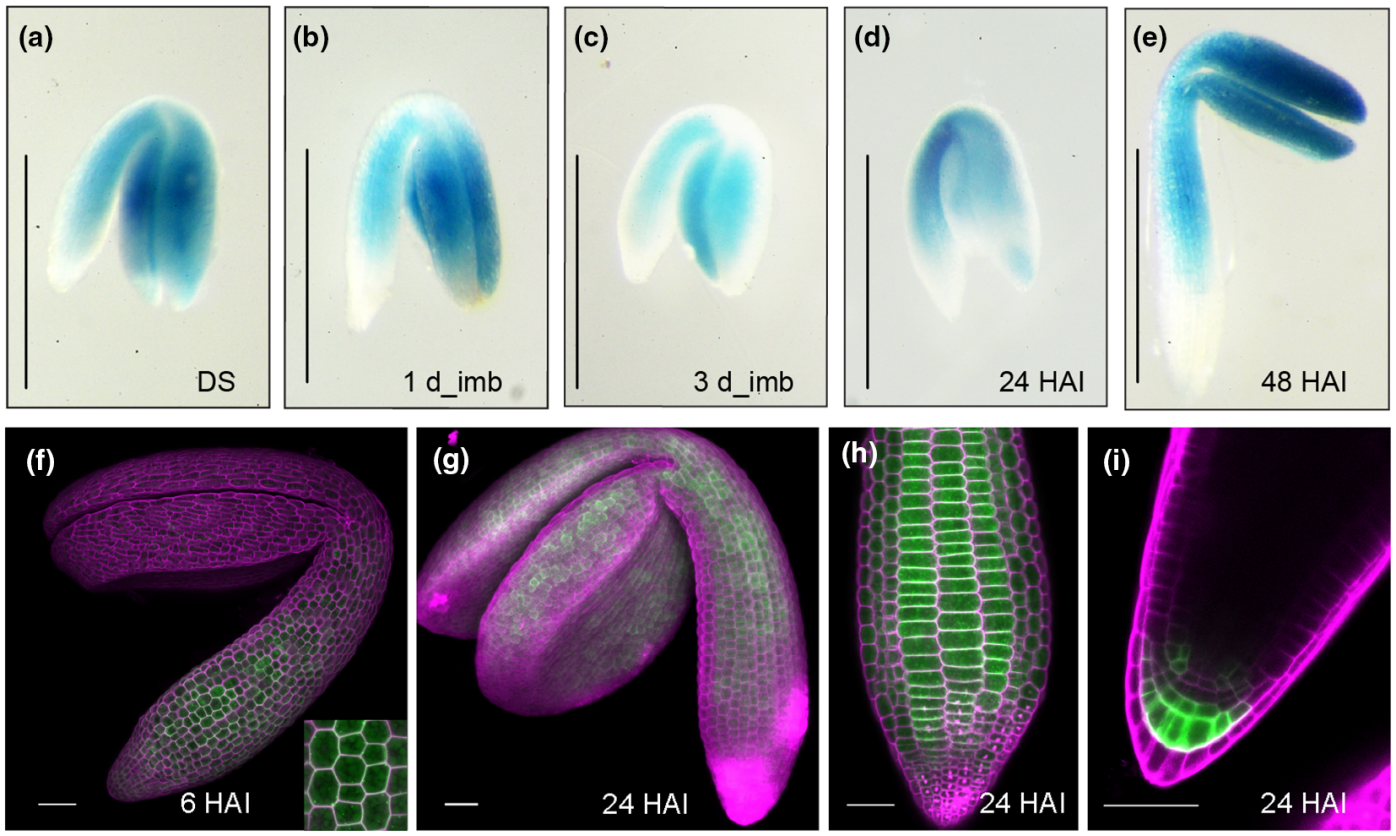
Expression profile of *SWEET1*. (a–e) SWEET1 accumulation pattern from dry seeds (DS) to seedlings. GUS staining of *pSWEET1:gSWEET1‐GUS* was observed in dry seeds (a), imbibed seeds (b, c), and germinating seeds (d, e) at the indicated stages after imbibition. Bars, 0.5 mm. (f) SWEET1‐YFP is detected in the epidermis of hypocotyl at 6 h after imbibition (HAI). The inset shows an enlarged hypocotyl epidermal cell. Bar, 50 μm. (g–i) SWEET1 is detectable in germinating seeds. SWEET1‐YFP under control of native promoter is active in cotyledon (g), cortex cells of the hypocotyl (h), and root tip (i) at 24 HAI. Bars, 50 μm. GUS, β‐glucuronidase; YFP, yellow fluorescent protein.

To determine whether SWEET1 is potentially involved in sugar allocation among different tissues, we analyzed its tissue expression pattern throughout the life cycle. The SWEET1‐GUS translational reporter line was generated by fusing the genomic coding region with *GUS* under the control of the native promoter (*pSWEET1:gSWEET1‐GUS*). We observed that SWEET1 was active in dry seeds and remained high in the whole embryo except for the radical tip, proceeding from imbibition to 2 DAI, and then appeared to accumulate in the root tip and newly emerging true leaves until 4 DAI (Figs 3a–e, S3a–c). There was no GUS staining detected in the endosperm and testa (Fig. S3a). In the first pair of true leaves, GUS signals appeared on the veins (Fig. S3d).

Since cell elongation in the hypocotyl is critical for germination and SWEET1‐GUS shows interesting patterns before and post‐germination, we in turn investigate the cell‐type expression pattern in *pSWEET1:gSWEET1‐YFP*, *sweet1* plants. SWEET1‐YFP was detected first in hypocotyl epidermal cells (Fig. 3f), as well as weak expression in cortex cells at 6 HAI (Fig. S3e). Then SWEET1‐YFP was expressed in the whole embryo at 24 HAI (Fig. 3g) and strong expression was found in hypocotyl cortex cells (Fig. 3h) and root tip, relatively stronger in cortex/endodermis initials, epidermis/lateral root cap initials, and columella cells (Fig. 3i). Because SWEET1‐YFP accumulates at the root tip and SWEET1 mediates sugar allocation, we speculate that SWEET1 may contribute to root development.

### Sugar suppression of ABA inhibition is dependent on the availability of substrate and the corresponding transporters locally

To explore the possible mechanism underlying SWEET1 being involved in seed germination under ABA + Glc, we evaluated the *SWEET1* transcript and protein levels throughout seed germination at different time points upon treatment. Our quantitative polymerase chain reaction revealed that the *SWEET1* transcript was slightly upregulated by Glc at 12 HAI, showed no change at 24 HAI, whereas it was upregulated by Glc, ABA, and ABA + Glc at 48 HAI (Fig. S4a).

**Fig. 4:**
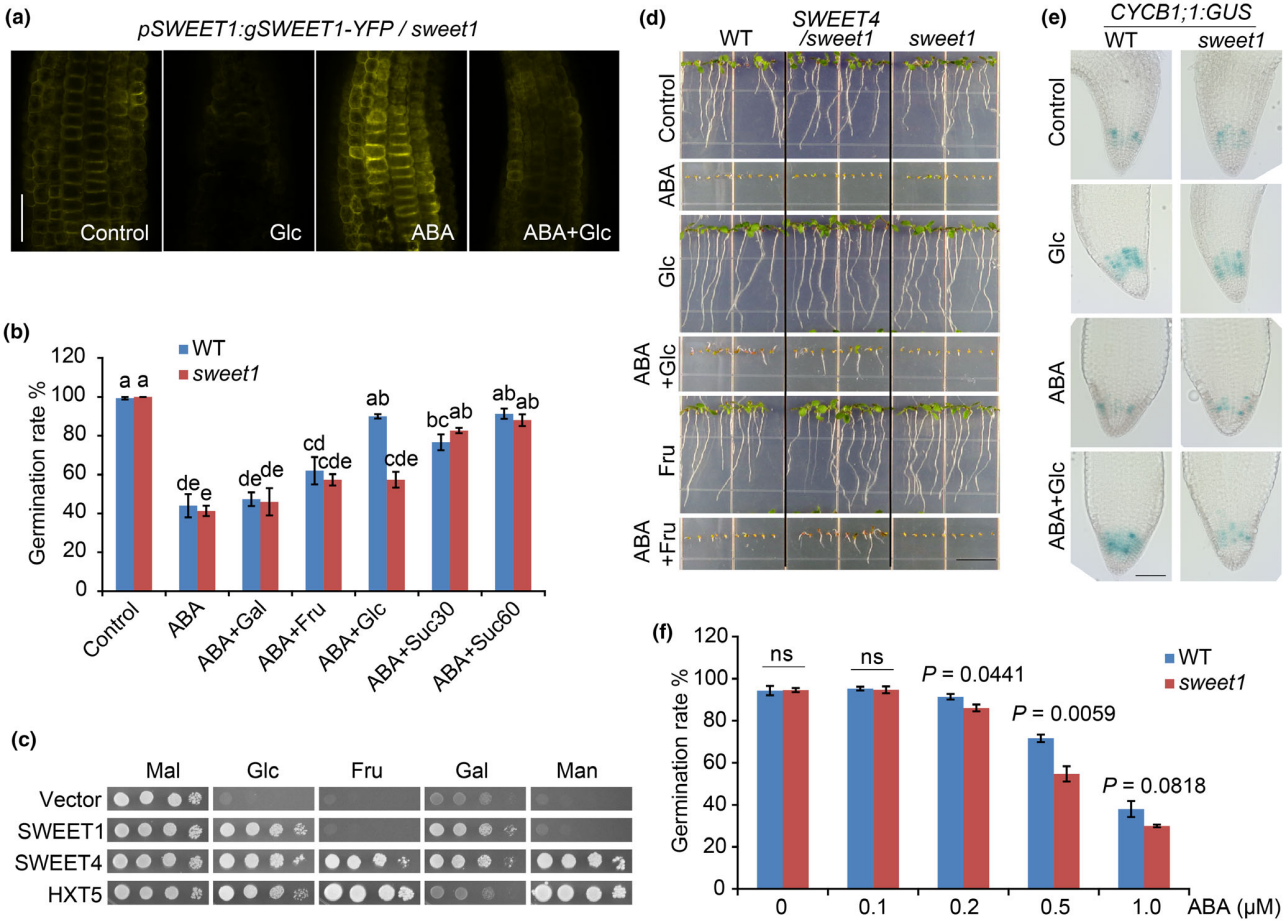
Sugar relief of abscisic acid (ABA) inhibition depends on transporter activities. (a) Glucose (Glc) and ABA regulate SWEET1 abundance in the hypocotyl during germination. The *pSWEET1:gSWEET1‐YFP/sweet1* seeds were sown on a ½‐strength Murashige & Skoog medium (½MS) medium supplemented with Glc, ABA, and ABA + Glc, respectively. Lower SWEET1‐YFP levels were detected under Glc, and higher SWEET1‐YFP levels were observed under ABA in the hypocotyl at 24 h after imbibition (HAI). Bar, 50 μm. (b) Germination rate of WT and *sweet1* seeds under ABA or ABA + sugar treatment. After a 3‐d imbibition, seeds were sown on ½MS medium supplemented with ABA or ABA with 60 mM of a hexose, namely galactose (Gal), fructose (Fru), and Glc, or sucrose (Suc) at two concentrations. The germination rates at 72 HAI were shown as means ± SE (*n* = 3). Different lowercase letters indicate significantly different means at *P* < 0.05 by one‐way ANOVA Tukey test. (c) Transport activities of SWEET1 and SWEET4 in yeast. The complementation assay was performed in yeast strain EBY.VW4000. Yeast transformed with Arabidopsis SWEET1, SWEET4, yeast hexose transporter HXT5, or empty vector (vector) were cultured on the medium supplemented with 2% of sugar, namely maltose (Mal), Glc, Fru, Gal, and mannose (Man). SWEET4 can transport Glc, Fru, Gal, and Man in yeast. (d) Root growth phenotypes of WT, *sweet1,* and *pSWEET1:gSWEET4‐YFP/sweet1* plants grown under ABA and ABA + sugar treatment. Both Glc and Fru can promote root growth under ABA conditions in *pSWEET1:gSWEET4‐YFP/sweet1* plants. Bar, 10 mm. (e) CYCB1;1‐GUS activities upon treatments. Seeds of CYCB1;1‐GUS in WT and *sweet1* were incubated on medium supplemented with Glc, ABA, or ABA + Glc for 48 h. Bar, 0.1 mm. (f) Germination rates of WT and sweet1 under low ABA concentrations at 48 h after imbibition. *P* values (Student's *t*‐test) are shown above the histogram. Values are means ± SE (*n* = 4). ns, no significant difference.

To test how SWEET1 responds to treatments during germination and if the reduced Glc uptake under ABA is due to the degradation of SWEET1, we used confocal microscopy to visualize the dynamics of SWEET1‐YFP driven by the *SWEET1* native promoter under treatments at 24 HAI. We found that in hypocotyl cortical cells, SWEET1‐YFP abundance was lowered by Glc but enhanced by ABA (Fig. 4a). Because SWEET1 transcript levels were not changed by Glc or ABA at 24 HAI (Fig. S4a), we speculated that the abundance of SWEET1‐YFP was affected by ABA and Glc at translational or posttranslational levels. In addition, the SWEET1‐YFP did show plasma membrane (PM) localization in germinating seeds, which is consistent with constitutively expressed SWEET1 (Chen *et al*., 2010).

The observation aforementioned supported that Glc suppressing ABA inhibition is unlikely to be explained by SWEET1 protein levels because SWEET1‐YFP level under ABA + Glc was lower than that under ABA (Fig. 4a). Then, we examined whether this process was associated with SWEET1 substrates. We tested the germination rate and primary root growth on a medium supplemented with ABA and other sugars. Interestingly, in *sweet1*, Suc could relieve ABA inhibition during both seed germination and seedling root growth, while Glc could not (Figs 4b, S4b). These data suggest that there must be another active Suc transporter, rather than SWEET1, that plays a primary role in Suc‐mediated relief of ABA inhibition, although SWEET1 shows weak Suc transporting activity (Chen *et al*., 2010). In addition, galactose (Gal) and mannitol (Mann) failed to relieve ABA inhibition on germination and root growth, respectively (Figs 4b, S4b).

It is worth noting that Fru only showed a slight rescue of germination and root growth in both WT and *sweet1* in the presence of ABA (Figs 4b, S4b), but was not significantly different from ABA treatment alone, which may be due to the lack of an efficient Fru transporter in germinating seeds. To test this notion and if either the transporter or a substrate of the transporter matters most, we introduced SWEET4 into our assay since SWEET4 can transport Fru (Liu *et al*., 2016) and its transcript was undetectable in germinating seeds (Fig. S1a). Before we introduced SWEET4 into the *sweet1* mutant, we tested the sugar transport activities of SWEET1 and SWEET4 in yeast. SWEET4 can transport Fru, Gal, and mannose (Man) in addition to Glc in yeast, showing the same activity as the positive control HXT5, whereas SWEET1 can transport Gal and Glc in our system (Fig. 4c). Then, we made a chimeric construct that replaced the *SWEET1* exons with the corresponding *SWEET4* exons, but kept the *SWEET1* introns and its promoter for a genetic complementation assay. As shown in Fig. 4(d), *sweet1* carrying *pSWEET1:gSWEET4‐YFP* displayed a phenotype comparable to that of WT when germinating on ABA + Glc medium, which indicates that the substrate transported by a transporter, not the transporter itself, matters most. It is worth noting that SWEET4 is expected to be a low‐affinity transporter, since all SWEETs with a measured *K*_m_ from Arabidopsis or other species have been demonstrated to be low‐affinity STPs (Chen *et al*., 2010, 2012; Guo *et al*., 2014; Ho *et al*., 2019; Xue *et al*., 2022). In the presence of Fru, seeds of *pSWEET1:SWEET4‐YFP/sweet1* germinated better on ABA medium than WT. These data suggest that the potential of a metabolizable sugar to antagonize ABA signaling is determined by substrate availability and activities of corresponding local transporters during seed germination.

### Sugar partitioning is compromised in *sweet1* during germination

It has been reported that Glc can activate the transcription of S‐phase‐related genes to promote cell proliferation in the root meristem zone (Xiong *et al*., 2013), while our data found that Glc evoked germination and primary root growth in the presence of ABA. To investigate whether Glc‐promoted cell division is compromised in *sweet1*, we introduced the cell cycle marker CYCB1;1‐GUS into the *sweet1* mutant and tested the activities upon treatments. GUS staining results showed that cell division was enhanced by Glc in both WT and *sweet1* at 48 HAI but suppressed by ABA in germinating seeds. This inhibition was also reversed by Glc addition in WT, but not in *sweet1* (Fig. 4e). These data suggest that under normal growth conditions, other sugar transporters such as STPs can compensate for the loss of SWEET1. However, in the presence of ABA, which inhibits other STPs, the loss of SWEET1 leads to Glc transport defects, thereby hindering root tip cell division. This phenomenon, namely that SWEET1 is conditionally functional, is consistent with the findings shown in Fig. 2(b,c).

Since SWEET1 functions when other STPs were inhibited by the presence of ABA, we needed to create a condition that met two criteria simultaneously to observe SWEET1 function without the addition of Glc. First, the ABA concentration must reach a level that inhibits STPs, and second, the ABA concentration must not be so high that the endogenous Glc level falls far below the *K*_m_ of SWEET1. Our previous study also found that germination inhibition caused by 2 μM ABA can be overcome by exogenous Glc (15–90 mM) in a dosage‐dependent manner (Xue *et al*., 2021). It is conceivable that endogenous Glc concentrations antagonize relatively low ABA levels. The lack of any seed germination phenotype for *sweet1* under normal conditions indicates that endogenous ABA levels may be below the antagonism window. One would assume that Glc may not be able to reach the antagonism window in a particular embryo subdomain in *sweet1* due to insufficient Glc allocation if a low amount of ABA is provided exogenously, which may allow us to observe any phenotypic differences between WT and *sweet1*. To test this assumption, we germinated *sweet1* at low ABA concentrations, from 0.1 to 1 μM. At these low ABA concentrations and without exogenous Glc, the *sweet1* mutation delayed germination compared to WT (Fig. 4f), although the *sweet1* phenotype was more severe when germinating on 2 μM ABA plus 60 mM Glc (Fig. 1d). This finding suggests that less sugar is partitioned to where it can suppress ABA inhibition in the *sweet1* mutant. However, it cannot be excluded that ABA signaling may be altered in the *sweet1* mutant.

### Glc antagonism of ABA response is globally weakened in *sweet1*

**Fig. 5:**
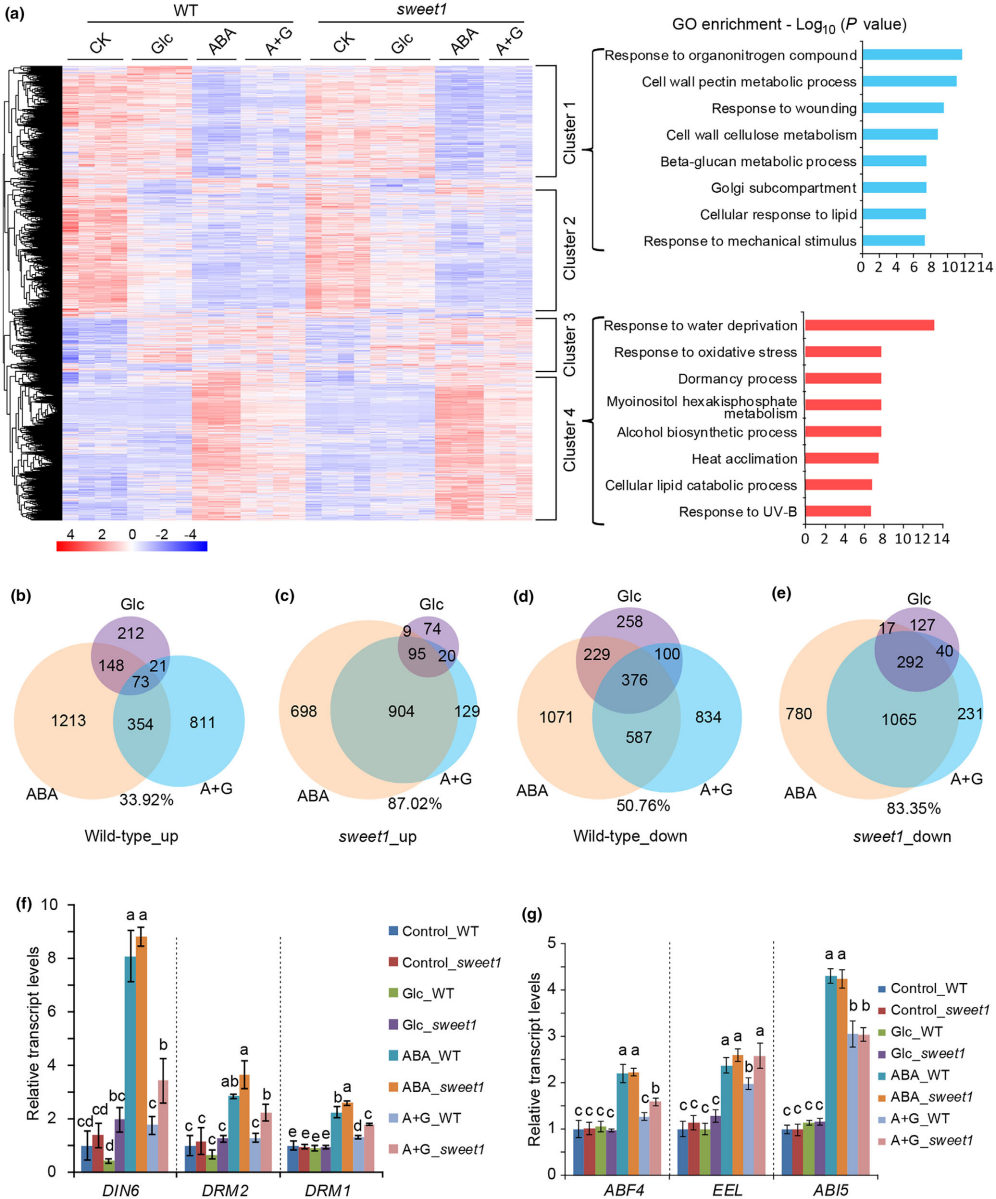
Glucose (Glc) antagonism of the abscisic acid (ABA) response is globally compromised in *sweet1*. (a) Heatmap of differentially expressed genes (DEGs). Four clusters of DEGs were classified based on the co‐expression pattern. The eight most enriched Gene Ontology terms in clusters 1 and 4 are presented in brackets. (b–e) The numbers of genes responding to Glc, ABA, and ABA + Glc in wild‐type (WT) and *sweet1*. The Venn diagrams represent DEGs (|*b*| ≥ 0.2, *q* < 0.05) with increased response upon treatments compared with that of the control condition in WT (b) and *sweet1* (c). (d, e) The numbers of downregulated DEGs upon treatments in WT (d) and *sweet1* (e), respectively. The percentage of ABA + Glc‐responsive genes overlapping with ABA‐responsive ones is shown below the diagrams. The overlapping ratio is higher in the *sweet1* background. (f) Transcript levels of SnRK1 activity marker genes. SnRK1 marker genes *DIN6*, *DRM1*, and *DRM2* were induced by ABA and suppressed by the addition of Glc. The transcript levels were higher in *sweet1* than in WT under ABA + Glc condition. Values are means ± SD (*n* = 3 or 4). (g) Transcript levels of bZIP genes under treatments. *ABF4*, *EEL*, and *ABI5* were induced by ABA. The expression of *ABF4* and *EEL* was reduced by the addition of Glc in WT, and to a lesser degree in *sweet1*, but *ABI5* was not. Values are means ± SD (*n* = 3 or 4). Different lowercase letters in (f, g) indicate significantly different means for each gene at *P* < 0.05 by one‐way ANOVA Tukey test.

Glc functions as a source of energy and carbon skeleton, as well as a signal molecule that regulates many biological processes and interplays with other signaling pathways, including ABA (Zhang *et al*., 2016; Wang *et al*., 2018; Fu *et al*., 2020). To better understand the molecular interaction between Glc and ABA and explain the *sweet1* germination phenotype, we performed transcriptome analysis comparing the downstream response genes in WT and *sweet1* under Glc, ABA, or ABA + Glc treatments. Pairwise comparison identified 6230 DEGs, which are grouped into four clusters (Fig. 5a; Dataset S1).

Gene ontology (GO) analysis revealed distinct functional enrichment in each cluster. Cluster 1 genes, suppressed by ABA and to a lesser degree by ABA + Glc, are involved in biological processes associated with response to organonitrogen compounds, cell wall pectin metabolism, wound response, and beta‐glucan metabolism. The genes in cluster 4 which were induced by ABA and, to a lesser degree, by ABA + Glc are linked to water deprivation, oxidative stress and heat, as well as processes like dormancy including genes *DRM1* (*dormancy‐associated gene 1*) and *DRM2* (*dormancy associated gene 2*), myoinositol hexakisphosphate metabolism, alcohol biosynthesis, and cellular lipid catabolism (Fig. 5a). Cluster 2 genes were suppressed by Glc, ABA, and ABA + Glc and were predicted to play roles in photosynthesis and amino acid metabolic processes. Cluster 3 genes were upregulated across all treatments and were predicted to function in gene regulation and the meiotic cell cycle (Dataset S2). As clusters 2 and 3 are uniformly regulated by the three treatments, they were not the focus of this study.

Upon Glc treatment, WT showed 454 upregulated genes, whereas *sweet1* had only 198 (Fig. 5b,c), and also fewer downregulated genes were observed in *sweet1* (Fig. 5d,e), consistent with the reduced Glc uptake in *sweet1* (Fig. 2b,c). Upon ABA + Glc treatment, 3156 DEGs were identified in WT and 2776 in *sweet1*. Among these, 44% overlapped with ABA‐responsive genes in WT, while this overlap rose to 85% in *sweet1* (Fig. 5b–e), indicating the transcriptomic response in *sweet1* under ABA + Glc is more similar to that under ABA alone. This pattern mirrors the germination phenotype, where Glc partially rescues ABA‐induced inhibition in WT but not in *sweet1* (Fig. 1d). More than 77% of the ABA‐inducible genes (FC ≥ 2, TPM ≥ 1.5) were suppressed by ABA + Glc in WT, but only *c*. 65% in *sweet1* (Fig. S5a). One example demonstration is that the transcript level of ABA‐responsive gene *RAB18* was downregulated in WT but upregulated in *sweet1* by Glc (Fig. S5b). *RAB18* was suppressed by ABA + Glc compared to ABA treatment in both *sweet1* and WT, but to a lesser degree in *sweet1*. These data indicate that ABA signaling is enhanced in the *sweet1* mutant in the presence of Glc.

### Transcript levels of genes in the SnRK1 pathway were enhanced in *sweet1* under ABA + Glc treatment

ABA application has been found to limit Glc availability in germinating seeds (Xue *et al*., 2021), while enhanced SnRK1 activity caused delayed germination (Tsai & Gazzarrini, 2012). Thus, we hypothesized that (1) ABA‐triggered Glc restriction would result in energy deprivation, which in turn activates an energy‐starvation signaling pathway mediated by SnRK1 (Rodriguez *et al*., 2019); (2) this pathway would, if active, in *sweet1* cannot be overcome by the presence of Glc since Glc fails to rescue the germination defect in *sweet1* (Fig. 1d). To test these hypotheses, we first surveyed the transcript levels of several marker genes that are downstream of KIN10/SnRK1.1, a kinase subunit of the SnRK1 complex. The transcript levels of SnRK1‐regulated genes, such as *DIN6* (dark inducible 6), *DRM1* and *DRM2,* were upregulated in *sweet1* compared to those in the WT under ABA + Glc (Fig. 5f), suggesting that the SnRK1 signaling pathway is more active in *sweet1* than in WT under ABA + Glc treatment. However, few of the SnRK1 complex genes showed significant differences among treatments or between genotypes (Fig. S6a). The genes *KING1* and *KING2* encoding the regulatory gamma subunits of the SnRK1 complex were upregulated by ABA and downregulated by ABA + Glc relative to ABA, but the changes were similar between *sweet1* and WT (Fig. S6a).

Since there were no significant differences in the transcript levels of genes encoding SnRK1 core components, we then expanded our survey list to include interactors of KIN10 because several basic leucine zipper (bZIP) transcription factors have been shown to physically interact with KIN10 and transduce the KIN10‐mediated primary signal for responses (Baena‐Gonzalez *et al*., 2007; Carianopol *et al*., 2020). Remarkably, *GBF1* (G‐BOX BINDING FACTOR 1), *GBF2*, *GBF3*, *ABF4* (ABRE BINDING FACTOR 4), *EEL* (ENHANCED EM LEVEL), and *ABI5* were upregulated by ABA but to a lesser degree by ABA + Glc in both WT and *sweet1* (Figs 5g, S6b). Among these genes, only *ABF4* and *EEL* were differentially expressed in *sweet1* and WT under ABA + Glc (Fig. 5g). This pattern aligns with the observation that Glc suppresses 130 of 139 ABA‐activated genes (FC ≥ 3) in WT (Xue *et al*., 2021), indicating Glc‐mediated targeting of key ABA‐regulated genes. Given their responsiveness, ABI5 and its homologs ABF4 and EEL are potential regulators under ABA + Glc conditions.

Since ABI5 plays an important role during ABA‐triggered germination suppression (Lopez‐Molina *et al*., 2002; Albertos *et al*., 2015), we tested *abi5‐8* germination rate as well as the ABI5 protein levels in WT and *sweet1* under our conditions. We found that the *abi5‐8* mutant was insensitive to ABA and ABA + Glc treatment (Fig. S7a). In addition, ABI5 protein was induced to a comparable level by ABA and ABA + Glc in both WT and *sweet1* (Fig. S7b). These data implied that ABI5 plays a role in the process of germination inhibition, but not in the Glc relieving germination inhibition caused by ABA via SWEET1. Taken together, these results indicate that the SnRK1‐integrated energy‐starvation signaling pathway may be involved in Glc suppression of ABA inhibition on seed germination through bZIP transcription factors, likely ABF4 and EEL, which requires further exploration.

### KIN10 functions in relieving the inhibition caused by ABA during seed germination

**Fig. 6:**
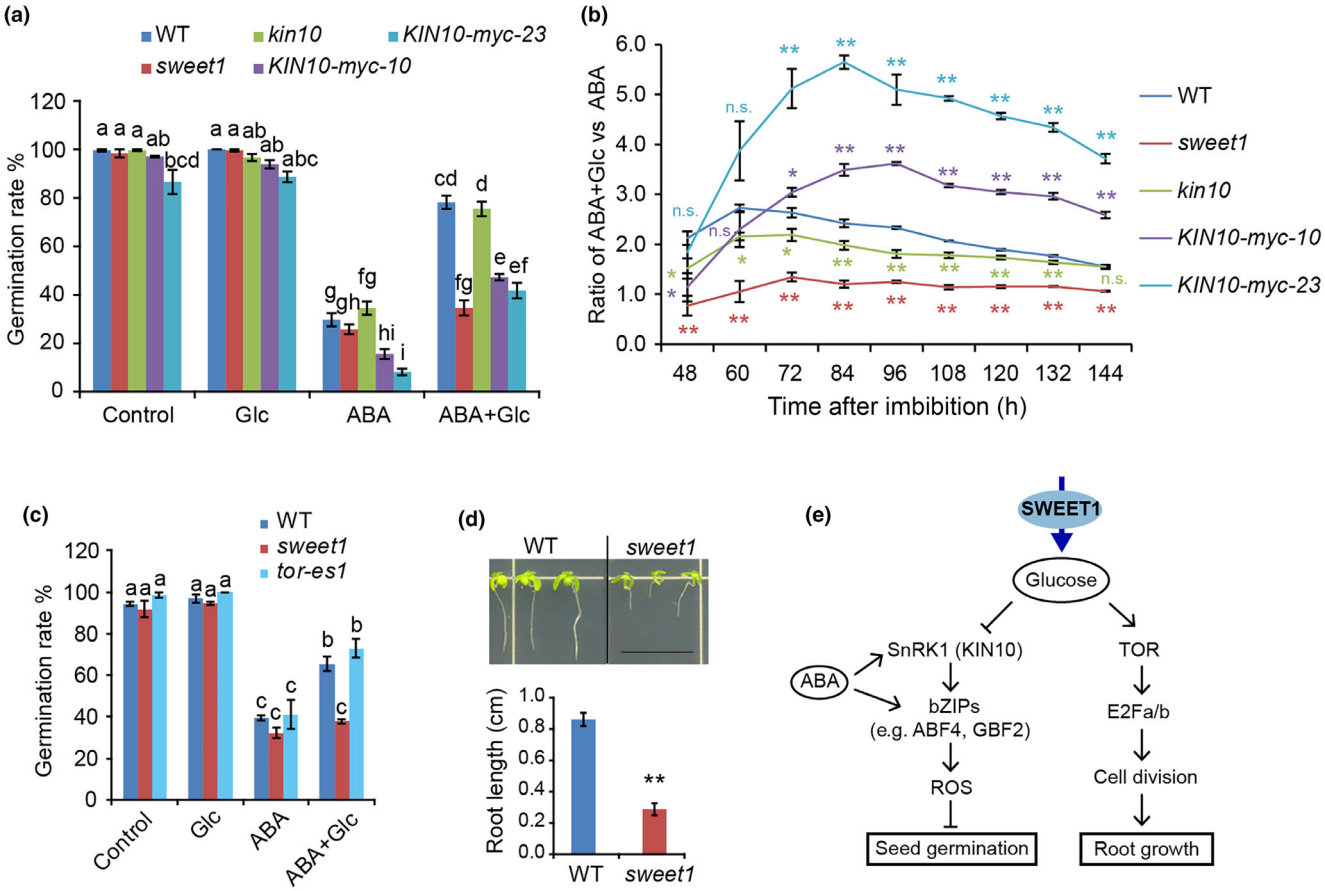
KIN10 functions in glucose (Glc)‐mediated suppression of the abscisic acid (ABA) inhibition. (a) Germination rates of the indicated plants under treatments at 72 hours after imbibition (HAI). Under ABA treatment, *kin10* germinated slightly earlier, while *KIN10* overexpression seeds germinated later. Values are means ± SE (*n* = 4). (b) The relative germination changes of the indicated plants. The ratio of germination rate on ABA + Glc vs that on ABA in *kin10* is always lower than that in WT during germination. Values are means ± SE (*n* = 4). Asterisks indicate significant differences compared with wild‐type (WT) values (Student's *t*‐test, *, *P* < 0.05; **, *P* < 0.01). ns, no significant difference. (c) Germination rate of *tor‐es1* under the treatments. Values are means ± SE (*n* = 3) at 60 HAI. Estradiol (10 μM) was added to the medium to induce *TOR* RNAi. Different lowercase letters in (a, c) indicate significantly different means at *P* < 0.05 by one‐way ANOVA Tukey test. (d) Glc fails to reactivate the arrested root growth in *sweet1*. The mitotically quiescent seedlings at 5 d after imbibition were transferred to medium supplemented with 15 mM Glc to grow for 8 d (top panel), and the quantification of the primary root length is shown in the bottom panel. Values are means ± SE (*n* = 8). Asterisks indicate significantly different compared with WT values (Student's *t*‐test, **, *P* < 0.01). Bar, 10 mm. (e) A working model for how SWEET1 functions in relieving ABA inhibition on germination in Arabidopsis. SWEET1‐mediated Glc inhibits the SnRK1 activities and therefore suppresses SnRK1‐ABA shared downstream targets, producing an ABA‐antagonistic effect in germinating seeds. After germination, Glc reactivates the arrested root growth via TOR‐directed cell division. Lines with an arrow indicate promotion, while those with a perpendicular bar indicate repression. ROS, reactive oxygen species; TOR, target of rapamycin.

To investigate the role of the SnRK1‐integrated energy‐starvation signaling pathway in antagonizing the effect of Glc on ABA, although *KIN10* is not regulated at the transcriptional level, we directly tested whether KIN10 functions in relieving Glc‐mediated ABA inhibition genetically. The *kin10* (GABI_579_E09) has been used as a KIN10 knock‐out mutant in studies characterizing its functions in low‐energy response, hypocotyl elongation, and stomatal development in plants (Mair *et al*., 2015; Simon *et al*., 2018; Ramon *et al*., 2019; Han *et al*., 2020). We observed that the *kin10* mutant germinates slightly earlier than WT under ABA treatment, but there is no significant difference (Fig. 6a). KIN10 overexpression (*p35S:KIN10‐myc*) displayed suppressed germination under our conditions (Fig. 6a), which aligns with a previous finding (Tsai & Gazzarrini, 2012). To assess the effects of KIN10 on ABA response in the presence of Glc, we compared the relative change in germination (ratio of ABA + Glc vs ABA) among WT, *sweet1*, *kin10*, and *KIN10* overexpression lines. The *kin10* consistently showed lower relative change than WT during germination, while the two independent overexpression lines showed an opposite trend across all tested time points except for 48 HAI (Fig. 6b), supporting that KIN10 contributes to the antagonistic effect of Glc on ABA during germination.

### SWEET1‐mediated Glc transport is involved in root meristem activation in postgerminative growth

ABA signaling in seedlings was suppressed by Glc‐activated TOR kinase that phosphorylates ABA receptors under optimal conditions (Wang *et al*., 2018). Therefore, we investigated whether the TOR pathway also contributes to Glc suppression of ABA inhibition on seed germination. After induction by 10 μM estradiol, the *TOR* RNAi mutant *tor‐es1* showed stunted growth of cotyledons, true leaves, petioles, and roots (Fig. S8a), the same as reported (Xiong & Sheen, 2012). However, two independent RNAi mutant lines, *tor‐es1* and *tor‐es2*, showed a comparable germination rate with WT and *sweet1* on ½MS medium (Fig. S8b). The antagonistic effect of Glc on ABA responses was not compromised in *tor‐e*s mutants (Figs 6c, S8c,d). This excludes TOR from participating in this process during germination.

Although TOR has differential functions in germinating seeds and seedlings, we wonder if SWEET1 shares similar functions in both stages. Reactivation of root growth under sugar starvation with the addition of exogenous Glc has been used to characterize TOR function in the seedling stage (Xiong *et al*., 2013). In root meristems, TOR phosphorylates transcription factor E2Fa, activating S‐phase genes to promote cell proliferation (Xiong *et al*., 2013). In our study, we found that exogenous Glc did not stimulate the primary root growth of *sweet1* as much as that of WT after sugar depletion (Fig. 6d), a phenotype that phenocopied *tor‐es* in response to exogenous Glc application (Xiong *et al*., 2013). Overall, our results suggest that SWEET1 also functions in postgerminative growth and is involved in root meristem activation, likely mediated by TOR signaling, which is different from the SnRK1‐signaling pathway involved in the role of SWEET1 during germination.

## Discussion

### SWEET1 is responsible for absorbing Glc under adverse conditions

ABA inhibits seed germination in part by reducing Glc uptake and limiting its Glc availability, especially in the hypocotyl region (Xue *et al*., 2021). Enzyme‐catalyzed sugar metabolism contributes to the sugar availability under ABA treatment. For example, the *sps* mutants with elevated Glc levels germinate earlier under ABA, which supports the notion that endogenous Glc antagonizes ABA inhibition *in vivo,* similar to exogenous Glc (Finkelstein & Lynch, 2000; Price *et al*., 2003; Xue *et al*., 2021). However, it remained elusive whether sugar allocation contributed to this Glc antagonism of ABA‐induced inhibition of germination. Here, we show that SWEET1 plays a key role in Glc allocation under ABA treatment. Mutation of *SWEET1* significantly impairs Glc uptake and prevents exogenous Glc from rescuing germination inhibition by ABA, suggesting that SWEET1‐mediated Glc transport is required for Glc to antagonize ABA (Fig. 1b,c). Although two other transporters, *STP1* and *STP4*, were suppressed by both ABA and Glc in *sweet1*, *sweet1* uptook a similar amount of Glc under both Glc and ABA + Glc treatments, indicating downregulation of *STPs* had limited impact in the presence of sufficient Glc (Fig. 2b). The remaining Glc uptake in *sweet1* may be attributed to other transporters, such as the PLTs. Although the absorbed Glc is sufficient to suppress the expression of *STP1* and *STP4* in *sweet1*, it is still not sufficient to overcome the germination inhibition caused by 2 μM ABA (Fig. 1d).

Based on transporter expression patterns, transporter activities, and affinities, we proposed, in the presence of exogenous Glc, the rank of Glc uptake capacity in germinating seeds is SWEET1 > PLTs > STPs. SWEET1 is a low‐affinity (*c*. 9 mM) Glc uniporter (Chen *et al*., 2010), whereas STP1 and STP4 have high affinities of 20 μM (Sherson *et al*., 2000) and 15 μM (Truernit *et al*., 1996), respectively. Under low external Glc, STPs are likely more efficient, as seen in Fig. 2(c), where Glc uptake was reduced in both WT and *sweet1* by ABA when only radiolabeled Glc was provided. By contrast, under high Glc conditions, SWEET1 becomes the dominant transporter.

In early germination, when reserves are not fully mobilized – as indicated by low activity of glyoxylate cycle enzyme ICL (ISOCITRATE LYASE) (Eastmond *et al*., 2000) and the gluconeogenesis enzyme PCK1 (PHOSPHOENOLPYRUVATE CARBOXYKINASE1) (Penfield *et al*., 2004)—high‐affinity STPs may dominate. Under stress, ABA may induce secondary dormancy by downregulating STPs and slowing sugar partitioning (Buijs, 2020). At later stages, when soluble sugars increase, low‐affinity transporters like SWEET1 may take over. If unfavorable conditions occur at this point, seeds may no longer re‐enter dormancy but instead require efficient sugar distribution to counteract ABA and promote germination.

SWEET1 is able to both export Glc into the cell wall space and to take up Glc into the cell along the concentration gradient. This mechanism provides a means for Glc to be transported from sugar‐rich to sugar‐poor cells or tissues. This could be especially important when symplastic connectivity is restricted, limiting direct cytoplasmic sugar movement.

The Arabidopsis embryo is symplasmically isolated from the cotyledons, shoot apex, hypocotyl, and root (Kim & Zambryski, 2005). The endosperm, physically active but symplasmically isolated from the embryo, stores one‐tenth of the fatty acids present in the whole seed (Penfield *et al*., 2004). SWEET1 is expressed in the epidermal cells of the embryo so that it can take up Glc leaked from the endosperm or derived from sucrose that has been hydrolyzed in the apoplasmic space into the embryo. SWEET1 may also be involved in Glc transport from other parts of the embryo to the hypocotyl since there is a lack of Glc under ABA treatment (Xue *et al*., 2021), and hypocotyl elongation is responsible for the completion of seed germination (Sliwinska *et al*., 2009). This speculation is also aligned with plasma membrane localization and the relatively high level of SWEET1 in the cotyledons, a dominant storage organ for lipids, proteins, and sugars during germination.

### Glc suppresses the ABA‐activated SnRK1 pathway during seed germination

It had been shown that ABI5 is a master regulator that controls seed germination in the ABA‐dependent signaling pathway (Lopez‐Molina *et al*., 2002; Albertos *et al*., 2015; Jin *et al*., 2018). The *ABI5* expression level was reduced to the same level in both WT and *sweet1* by the addition of Glc compared to ABA only (Fig. 5g), and levels of ABI5 protein were induced to comparable levels in both WT and *sweet1* by ABA + Glc (Fig. S7b), indicating that ABI5 may contribute to Glc‐mediated ABA inhibition relief, but likely independent of SWEET1. Levels of ABI5 protein or activities, even other transcription factors, may be involved, and this needs to be investigated in the future.

Sugars play a pivotal role in regulating plant growth, development, and responses to energy stress (Rolland *et al*., 2006). The finding that metabolizable sugars, not structural sugars, can overcome ABA inhibition of seed germination indicates that energy‐signaling pathways are involved. It is known that Glc limitation activates KIN10‐/11‐mediated energy sensing (Baena‐Gonzalez *et al*., 2007) through sugar phosphates, such as glucose‐6‐phosphate (G6P) and trehalose‐6‐phosphate (T6P), which directly suppress SnRK1 activity in plants (Toroser *et al*., 2000; Nunes *et al*., 2013). When we survey the common downstream genes between ABA and SnRK1 signaling, *c*. 20% of KIN10 response genes are also regulated by ABA (Fig. S9a,b), including the upregulated bZIP genes *ABF4* and *GBF2* (Fig. S9a). ABF4, as a common downstream gene of ABA and SnRK1, has been shown to promote the production of reactive oxygen species, thereby inhibiting seed germination (Li *et al*., 2023). Under ABA + Glc conditions, the transcript levels of *ABF4* and another bZIP gene, *EEL*, were higher in *sweet1* (Fig. 5g), indicating that ABF4 and EEL are potential regulatory factors responsible for the phenotypic differences between WT and *sweet1* (Fig. 6e).

This study reveals a SWEET1‐SnRK1 module in which SWEET1 absorbs Glc on the membrane and imported Glc pushes more carbon to be converted into sugar phosphates, such as G6P and T6P, which in turn inhibit the SnRK1‐ABA shared signaling pathway (Zhang *et al*., 2009; Nunes *et al*., 2013), thus producing an ABA‐antagonistic effect in germinating seeds (Fig. 6e). Our findings also support that SWEET1‐mediated Glc allocation can restore root growth in sugar‐starvation plants, likely through the TOR‐directed root meristem activation pathway (Xiong *et al*., 2013) at postgerminative growth (Fig. 6e).

### Transporters guide signaling intensity

The *sweet1* mutant is an ideal genetic material to dissect the interaction between Glc and ABA under stress conditions during germination. Although Glc failed to relieve ABA inhibition in *sweet1* as it did in WT, treatment with Glc still suppressed germination in *sweet1* and WT at 36 HAI (Fig. 1d), which indicates that the threshold for Glc to be effective is different for these two physiological processes or that there may be different underlying mechanisms. The amount of Glc absorbed by the *sweet1* mutant was sufficient to delay germination, but not enough to trigger sugar signaling pathways to override ABA (2 μM) inhibition on 60 mM Glc medium. This finding proved that these two processes are controlled by different Glc transporters and/or signaling pathways. Indeed, it has been reported that germination inhibition caused by Glc is independent of the HXK1 and ABIs (Dekkers *et al*., 2004). Thus, Glc likely delays germination through an ABA‐independent pathway.

As shown in Fig. 4(b), Fru cannot release ABA germination inhibition as efficiently as Glc can, mainly due to a lack of an efficient Fru transporter in germinating seeds. These results reveal the importance of spatiotemporal expression of a competent STP for seeds in overcoming adversity. Since both Glc and Fru can facilitate seed germination under stress, SWEET4 may be a good target for engineering to improve seed vigor under the control of the *SWEET1* promoter, as SWEET4 can transport both Glc and Fru, and *pSWEET1:gSWEET4‐YFP/sweet1* seeds indeed performed better than WT in the presence of ABA plus Glc or Fru (Fig. 4d).

## Competing interests

None declared.

## Author contributions

L‐QC oversaw the project. XX and L‐QC conceived and designed the experiments. XX, JL, and Y‐CY conducted experiments. XX and L‐QC analyzed data and wrote the manuscript.

## Disclaimer

The New Phytologist Foundation remains neutral with regard to jurisdictional claims in maps and in any institutional affiliations.

## Supporting information


**Dataset S1** Oligonucleotides and DEG annotation.

**Dataset S2** GO enrichment in each cluster.

**Fig. S1** Transcript levels of *SWEET*, *STP*, and *PLT* families in germinating seeds.
**Fig. S2** Characterization of *sweet1* mutants.
**Fig. S3** Expression profile of *SWEET1*.
**Fig. S4** *SWEET1* levels under treatments and *sweet1* phenotypes respond to different sugars.
**Fig. S5** Compromised Glc response in *sweet1*.
**Fig. S6** Expression of SnRK1‐related genes upon treatments in germinating seeds.
**Fig. S7** ABI5 unlikely contributing to Glc antagonizing ABA inhibition on seed germination.
**Fig. S8** TOR lacking contribution to Glc suppression of ABA inhibition on seed germination.
**Fig. S9** Downstream genes shared by ABA and KIN10.
Please note: Wiley is not responsible for the content or functionality of any Supporting Information supplied by the authors. Any queries (other than missing material) should be directed to the *New Phytologist* Central Office.

## Data Availability

The RNA‐sequencing data that support the findings of this study are available in the National Center for Biotechnology Information GEO database (accession nos.: GSE163057 and GSE193111).

## References

[nph70738-bib-0001] Albertos P , Romero‐Puertas MC , Tatematsu K , Mateos I , Sanchez‐Vicente I , Nambara E , Lorenzo O . 2015. S‐nitrosylation triggers ABI5 degradation to promote seed germination and seedling growth. Nature Communications 6: 8669. 10.1038/ncomms9669 PMC4639896 26493030

[nph70738-bib-0002] Baena‐Gonzalez E , Rolland F , Thevelein JM , Sheen J . 2007. A central integrator of transcription networks in plant stress and energy signalling. Nature 448: 938–942. 17671505 10.1038/nature06069

[nph70738-bib-0003] Bewley JD . 1997. Seed germination and dormancy. Plant Cell 9: 1055–1066. 12237375 10.1105/tpc.9.7.1055 PMC156979

[nph70738-bib-0004] Bray NL , Pimentel H , Melsted P , Pachter L . 2016. Near‐optimal probabilistic RNA‐seq quantification. Nature Biotechnology 34: 525–527. 10.1038/nbt.3519 27043002

[nph70738-bib-0005] Buijs G . 2020. A perspective on secondary seed dormancy in *Arabidopsis thaliana* . Plants 9: 749. 32549219 10.3390/plants9060749 PMC7355504

[nph70738-bib-0006] Buttner M . 2010. The Arabidopsis sugar transporter (AtSTP) family: an update. Plant Biology 12(Suppl 1): 35–41. 10.1111/j.1438-8677.2010.00383.x 20712619

[nph70738-bib-0007] Carianopol CS , Chan AL , Dong S , Provart NJ , Lumba S , Gazzarrini S . 2020. An abscisic acid‐responsive protein interaction network for sucrose non‐fermenting related kinase1 in abiotic stress response. Communications Biology 3: 145. 32218501 10.1038/s42003-020-0866-8 PMC7099082

[nph70738-bib-0008] Chen JG , Willard FS , Huang J , Liang J , Chasse SA , Jones AM , Siderovski DP . 2003. A seven‐transmembrane RGS protein that modulates plant cell proliferation. Science 301: 1728–1731. 14500984 10.1126/science.1087790

[nph70738-bib-0009] Chen LQ , Hou BH , Lalonde S , Takanaga H , Hartung ML , Qu XQ , Guo WJ , Kim JG , Underwood W , Chaudhuri B *et al*. 2010. Sugar transporters for intercellular exchange and nutrition of pathogens. Nature 468: 527–532. 21107422 10.1038/nature09606 PMC3000469

[nph70738-bib-0010] Chen LQ , Lin IW , Qu XQ , Sosso D , McFarlane HE , Londono A , Samuels AL , Frommer WB . 2015. A cascade of sequentially expressed sucrose transporters in the seed coat and endosperm provides nutrition for the Arabidopsis embryo. Plant Cell 27: 607–619. 25794936 10.1105/tpc.114.134585 PMC4558658

[nph70738-bib-0011] Chen LQ , Qu XQ , Hou BH , Sosso D , Osorio S , Fernie AR , Frommer WB . 2012. Sucrose efflux mediated by SWEET proteins as a key step for phloem transport. Science 335: 207–211. 22157085 10.1126/science.1213351

[nph70738-bib-0012] Cutler SR , Rodriguez PL , Finkelstein RR , Abrams SR . 2010. Abscisic acid: emergence of a core signaling network. Annual Review of Plant Biology 61: 651–679. 10.1146/annurev-arplant-042809-112122 20192755

[nph70738-bib-0013] Dekkers BJ , Schuurmans JA , Smeekens SC . 2004. Glucose delays seed germination in *Arabidopsis thaliana* . Planta 218: 579–588. 14648119 10.1007/s00425-003-1154-9

[nph70738-bib-0014] Eastmond PJ , Germain V , Lange PR , Bryce JH , Smith SM , Graham IA . 2000. Postgerminative growth and lipid catabolism in oilseeds lacking the glyoxylate cycle. Proceedings of the National Academy of Sciences, USA 97: 5669–5674. 10.1073/pnas.97.10.5669 PMC25886 10805817

[nph70738-bib-0015] Finkelstein R , Reeves W , Ariizumi T , Steber C . 2008. Molecular aspects of seed dormancy. Annual Review of Plant Biology 59: 387–415. 10.1146/annurev.arplant.59.032607.092740 18257711

[nph70738-bib-0016] Finkelstein RR , Gampala SS , Rock CD . 2002. Abscisic acid signaling in seeds and seedlings. Plant Cell 14(Suppl): S15–S45. 12045268 10.1105/tpc.010441 PMC151246

[nph70738-bib-0017] Finkelstein RR , Lynch TJ . 2000. Abscisic acid inhibition of radicle emergence but not seedling growth is suppressed by sugars. Plant Physiology 122: 1179–1186. 10759513 10.1104/pp.122.4.1179 PMC58952

[nph70738-bib-0018] Fu L , Wang P , Xiong Y . 2020. Target of Rapamycin signaling in plant stress responses. Plant Physiology 182: 1613–1623. 31949028 10.1104/pp.19.01214 PMC7140942

[nph70738-bib-0019] Garciarrubio A , Legaria JP , Covarrubias AA . 1997. Abscisic acid inhibits germination of mature *Arabidopsis* seeds by limiting the availability of energy and nutrients. Planta 203: 182–187. 9362564 10.1007/s004250050180

[nph70738-bib-0020] Guo WJ , Nagy R , Chen HY , Pfrunder S , Yu YC , Santelia D , Frommer WB , Martinoia E . 2014. SWEET17, a facilitative transporter, mediates fructose transport across the tonoplast of Arabidopsis roots and leaves. Plant Physiology 164: 777–789. 24381066 10.1104/pp.113.232751 PMC3912105

[nph70738-bib-0021] Han C , Liu Y , Shi W , Qiao Y , Wang L , Tian Y , Fan M , Deng Z , Lau OS , De Jaeger G *et al*. 2020. KIN10 promotes stomatal development through stabilization of the SPEECHLESS transcription factor. Nature Communications 11: 4214. 10.1038/s41467-020-18048-w PMC7447634 32843632

[nph70738-bib-0022] Ho L‐H , Klemens PAW , Neuhaus HE , Ko H‐Y , Hsieh S‐Y , Guo W‐J . 2019. SlSWEET1a is involved in glucose import to young leaves in tomato plants. Journal of Experimental Botany 70: 3241–3254. 30958535 10.1093/jxb/erz154 PMC6598072

[nph70738-bib-0023] Hong SP , Carlson M . 2007. Regulation of snf1 protein kinase in response to environmental stress. The Journal of Biological Chemistry 282: 16838–16845. 17438333 10.1074/jbc.M700146200

[nph70738-bib-0024] Hu DG , Sun CH , Zhang QY , An JP , You CX , Hao YJ . 2016. Glucose sensor MdHXK1 phosphorylates and stabilizes MdbHLH3 to promote anthocyanin biosynthesis in apple. PLoS Genetics 12: e1006273. 27560976 10.1371/journal.pgen.1006273 PMC4999241

[nph70738-bib-0025] Jang JC , Leon P , Zhou L , Sheen J . 1997. Hexokinase as a sugar sensor in higher plants. Plant Cell 9: 5–19. 9014361 10.1105/tpc.9.1.5 PMC156897

[nph70738-bib-0026] Jin D , Wu M , Li B , Bucker B , Keil P , Zhang S , Li J , Kang D , Liu J , Dong J *et al*. 2018. The COP9 signalosome regulates seed germination by facilitating protein degradation of RGL2 and ABI5. PLoS Genetics 14: e1007237. 29462139 10.1371/journal.pgen.1007237 PMC5834205

[nph70738-bib-0027] Kang J , Yim S , Choi H , Kim A , Lee KP , Lopez‐Molina L , Martinoia E , Lee Y . 2015. Abscisic acid transporters cooperate to control seed germination. Nature Communications 6: 8113. 10.1038/ncomms9113 PMC4569717 26334616

[nph70738-bib-0028] Kim I , Zambryski PC . 2005. Cell‐to‐cell communication via plasmodesmata during *Arabidopsis* embryogenesis. Current Opinion in Plant Biology 8: 593–599. 16207533 10.1016/j.pbi.2005.09.013

[nph70738-bib-0029] Le Guen L , Thomas M , Bianchi M , Halford NG , Kreis M . 1992. Structure and expression of a gene from *Arabidopsis thaliana* encoding a protein related to SNF1 protein kinase. Gene 120: 249–254. 1339373 10.1016/0378-1119(92)90100-4

[nph70738-bib-0030] Lee KP , Piskurewicz U , Tureckova V , Strnad M , Lopez‐Molina L . 2010. A seed coat bedding assay shows that RGL2‐dependent release of abscisic acid by the endosperm controls embryo growth in *Arabidopsis* dormant seeds. Proceedings of the National Academy of Sciences, USA 107: 19108–19113. 10.1073/pnas.1012896107 PMC2973907 20956298

[nph70738-bib-0031] Li L , Li L , Cui S , Qian D , Lyu S , Liu W , Botella JR , Li H , Burritt DJ , Tran L‐SP *et al*. 2023. PDC1 is activated by ABF4 and inhibits seed germination by promoting ROS accumulation in Arabidopsis. Environmental and Experimental Botany 206: 105188.

[nph70738-bib-0032] Li Y , Lee KK , Walsh S , Smith C , Hadingham S , Sorefan K , Cawley G , Bevan MW . 2006. Establishing glucose‐ and ABA‐regulated transcription networks in *Arabidopsis* by microarray analysis and promoter classification using a Relevance Vector Machine. Genome Research 16: 414–427. 16424108 10.1101/gr.4237406 PMC1415219

[nph70738-bib-0033] Liu X , Zhang Y , Yang C , Tian Z , Li J . 2016. AtSWEET4, a hexose facilitator, mediates sugar transport to axial sinks and affects plant development. Scientific Reports 6: 24563. 27102826 10.1038/srep24563 PMC4840376

[nph70738-bib-0034] Lopez‐Molina L , Mongrand S , McLachlin DT , Chait BT , Chua NH . 2002. ABI5 acts downstream of ABI3 to execute an ABA‐dependent growth arrest during germination. The Plant Journal 32: 317–328. 12410810 10.1046/j.1365-313x.2002.01430.x

[nph70738-bib-0035] Mair A , Pedrotti L , Wurzinger B , Anrather D , Simeunovic A , Weiste C , Valerio C , Dietrich K , Kirchler T , Nagele T *et al*. 2015. SnRK1‐triggered switch of bZIP63 dimerization mediates the low‐energy response in plants. eLife 4: e05828. 26263501 10.7554/eLife.05828 PMC4558565

[nph70738-bib-0036] Menand B , Desnos T , Nussaume L , Berger F , Bouchez D , Meyer C , Robaglia C . 2002. Expression and disruption of the *Arabidopsis TOR* (target of rapamycin) gene. Proceedings of the National Academy of Sciences, USA 99: 6422–6427. 10.1073/pnas.092141899 PMC122964 11983923

[nph70738-bib-0037] Moore B , Zhou L , Rolland F , Hall Q , Cheng WH , Liu YX , Hwang I , Jones T , Sheen J . 2003. Role of the *Arabidopsis* glucose sensor HXK1 in nutrient, light, and hormonal signaling. Science 300: 332–336. 12690200 10.1126/science.1080585

[nph70738-bib-0038] Nunes C , Primavesi LF , Patel MK , Martinez‐Barajas E , Powers SJ , Sagar R , Fevereiro PS , Davis BG , Paul MJ . 2013. Inhibition of SnRK1 by metabolites: tissue‐dependent effects and cooperative inhibition by glucose 1‐phosphate in combination with trehalose 6‐phosphate. Plant Physiology and Biochemistry 63: 89–98. 23257075 10.1016/j.plaphy.2012.11.011

[nph70738-bib-0039] Penfield S , Rylott EL , Gilday AD , Graham S , Larson TR , Graham IA . 2004. Reserve mobilization in the Arabidopsis endosperm fuels hypocotyl elongation in the dark, is independent of abscisic acid, and requires PHOSPHOENOLPYRUVATE CARBOXYKINASE1. Plant Cell 16: 2705–2718. 15367715 10.1105/tpc.104.024711 PMC520966

[nph70738-bib-0040] Pimentel H , Bray NL , Puente S , Melsted P , Pachter L . 2017. Differential analysis of RNA‐seq incorporating quantification uncertainty. Nature Methods 14: 687–690. 28581496 10.1038/nmeth.4324

[nph70738-bib-0041] Price J , Li TC , Kang SG , Na JK , Jang JC . 2003. Mechanisms of glucose signaling during germination of Arabidopsis. Plant Physiology 132: 1424–1438. 12857824 10.1104/pp.103.020347 PMC167082

[nph70738-bib-0042] Quettier AL , Eastmond PJ . 2009. Storage oil hydrolysis during early seedling growth. Plant Physiology and Biochemistry 47: 485–490. 19136267 10.1016/j.plaphy.2008.12.005

[nph70738-bib-0043] Ramon M , Dang TVT , Broeckx T , Hulsmans S , Crepin N , Sheen J , Rolland F . 2019. Default activation and nuclear translocation of the plant cellular energy sensor SnRK1 regulate metabolic stress responses and development. Plant Cell 31: 1614–1632. 31123051 10.1105/tpc.18.00500 PMC6635846

[nph70738-bib-0044] Rodriguez M , Parola R , Andreola S , Pereyra C , Martinez‐Noel G . 2019. TOR and SnRK1 signaling pathways in plant response to abiotic stresses: do they always act according to the “yin‐yang” model? Plant Science 288: 110220. 31521220 10.1016/j.plantsci.2019.110220

[nph70738-bib-0045] Rolland F , Baena‐Gonzalez E , Sheen J . 2006. Sugar sensing and signaling in plants: conserved and novel mechanisms. Annual Review of Plant Biology 57: 675–709. 10.1146/annurev.arplant.57.032905.105441 16669778

[nph70738-bib-0046] Sanchez‐Montesino R , Bouza‐Morcillo L , Marquez J , Ghita M , Duran‐Nebreda S , Gomez L , Holdsworth MJ , Bassel G , Onate‐Sanchez L . 2019. A regulatory module controlling GA‐mediated endosperm cell expansion is critical for seed germination in *Arabidopsis* . Molecular Plant 12: 71–85. 30419294 10.1016/j.molp.2018.10.009 PMC7086157

[nph70738-bib-0047] Sherson SM , Hemmann G , Wallace G , Forbes S , Germain V , Stadler R , Bechtold N , Sauer N , Smith SM . 2000. Monosaccharide/proton symporter AtSTP1 plays a major role in uptake and response of *Arabidopsis* seeds and seedlings to sugars. The Plant Journal 24: 849–857. 11135118 10.1046/j.1365-313x.2000.00935.x

[nph70738-bib-0048] Simon NML , Sawkins E , Dodd AN . 2018. Involvement of the SnRK1 subunit KIN10 in sucrose‐induced hypocotyl elongation. Plant Signaling & Behavior 13: e1457913. 29584583 10.1080/15592324.2018.1457913 PMC6110359

[nph70738-bib-0049] Sliwinska E , Bassel GW , Bewley JD . 2009. Germination of *Arabidopsis thaliana* seeds is not completed as a result of elongation of the radicle but of the adjacent transition zone and lower hypocotyl. Journal of Experimental Botany 60: 3587–3594. 19620183 10.1093/jxb/erp203

[nph70738-bib-0050] Solfanelli C , Poggi A , Loreti E , Alpi A , Perata P . 2006. Sucrose‐specific induction of the anthocyanin biosynthetic pathway in Arabidopsis. Plant Physiology 140: 637–646. 16384906 10.1104/pp.105.072579 PMC1361330

[nph70738-bib-0051] Streb S , Zeeman SC . 2012. Starch metabolism in Arabidopsis. The Arabidopsis Book 10: e0160. 23393426 10.1199/tab.0160 PMC3527087

[nph70738-bib-0052] Toroser D , Plaut Z , Huber SC . 2000. Regulation of a plant SNF1‐related protein kinase by glucose‐6‐phosphate. Plant Physiology 123: 403–412. 10806257 10.1104/pp.123.1.403 PMC59014

[nph70738-bib-0053] Truernit E , Schmid J , Epple P , Illig J , Sauer N . 1996. The sink‐specific and stress‐regulated Arabidopsis STP4 gene: enhanced expression of a gene encoding a monosaccharide transporter by wounding, elicitors, and pathogen challenge. Plant Cell 8: 2169–2182. 8989877 10.1105/tpc.8.12.2169 PMC161343

[nph70738-bib-0054] Tsai AY , Gazzarrini S . 2012. AKIN10 and FUSCA3 interact to control lateral organ development and phase transitions in Arabidopsis. The Plant Journal 69: 809–821. 22026387 10.1111/j.1365-313X.2011.04832.x

[nph70738-bib-0055] Tuan PA , Kumar R , Rehal PK , Toora PK , Ayele BT . 2018. Molecular mechanisms underlying abscisic acid/gibberellin balance in the control of seed dormancy and germination in cereals. Frontiers in Plant Science 9: 668. 29875780 10.3389/fpls.2018.00668 PMC5974119

[nph70738-bib-0056] Wang P , Zhao Y , Li Z , Hsu CC , Liu X , Fu L , Hou YJ , Du Y , Xie S , Zhang C *et al*. 2018. Reciprocal regulation of the TOR kinase and ABA receptor balances plant growth and stress response. Molecular Cell 69: 100–112. 29290610 10.1016/j.molcel.2017.12.002 PMC5772982

[nph70738-bib-0057] Woods A , Munday MR , Scott J , Yang X , Carlson M , Carling D . 1994. Yeast SNF1 is functionally related to mammalian AMP‐activated protein kinase and regulates acetyl‐CoA carboxylase *in vivo* . The Journal of Biological Chemistry 269: 19509–19515. 7913470

[nph70738-bib-0058] Xiong Y , McCormack M , Li L , Hall Q , Xiang C , Sheen J . 2013. Glucose‐TOR signalling reprograms the transcriptome and activates meristems. Nature 496: 181–186. 23542588 10.1038/nature12030 PMC4140196

[nph70738-bib-0059] Xiong Y , Sheen J . 2012. Rapamycin and glucose‐target of rapamycin (TOR) protein signaling in plants. The Journal of Biological Chemistry 287: 2836–2842. 22134914 10.1074/jbc.M111.300749 PMC3268441

[nph70738-bib-0066] Xue X , Wang J , Shukla D , Cheung LS , Chen LQ . 2022. When SWEETs turn tweens: Updates and perspectives. Annual Review of Plant Biology 73: 379–403. 10.1146/annurev-arplant-070621-093907 34910586

[nph70738-bib-0060] Xue X , Yu Y‐C , Wu Y , Xue H , Chen L‐Q . 2021. Locally restricted glucose availability in the embryonic hypocotyl determines seed germination under ABA treatment. New Phytologist 231: 1832–1844. 34032290 10.1111/nph.17513

[nph70738-bib-0061] Yan L , Wei S , Wu Y , Hu R , Li H , Yang W , Xie Q . 2015. High‐efficiency genome editing in Arabidopsis using YAO promoter‐driven CRISPR/Cas9 system. Molecular Plant 8: 1820–1823. 26524930 10.1016/j.molp.2015.10.004

[nph70738-bib-0062] Zhang Y , Primavesi LF , Jhurreea D , Andralojc PJ , Mitchell RA , Powers SJ , Schluepmann H , Delatte T , Wingler A , Paul MJ . 2009. Inhibition of SNF1‐related protein kinase1 activity and regulation of metabolic pathways by trehalose‐6‐phosphate. Plant Physiology 149: 1860–1871. 19193861 10.1104/pp.108.133934 PMC2663748

[nph70738-bib-0063] Zhang Z , Zhu JY , Roh J , Marchive C , Kim SK , Meyer C , Sun Y , Wang W , Wang ZY . 2016. TOR signaling promotes accumulation of BZR1 to balance growth with carbon availability in Arabidopsis. Current Biology 26: 1854–1860. 27345161 10.1016/j.cub.2016.05.005 PMC5126233

[nph70738-bib-0064] Zhao M , Zhang H , Yan H , Qiu L , Baskin CC . 2018. Mobilization and role of starch, protein, and fat reserves during seed germination of six wild grassland species. Frontiers in Plant Science 9: 234. 29535748 10.3389/fpls.2018.00234 PMC5835038

[nph70738-bib-0065] Zhu JK . 2016. Abiotic stress signaling and responses in plants. Cell 167: 313–324. 27716505 10.1016/j.cell.2016.08.029 PMC5104190

