# Supplementary material for: SWEET1‐mediated glucose transport is crucial for energy availability in Arabidopsis
Source: New Phytol. 2025 Nov 18;249(4):1816–30. doi: 10.1111/nph.70738 (PMC12825402; doi:10.1111/nph.70738)
Supplement: Supplementary file 3 — Fig. S1 Transcript levels of SWEET, STP, and PLT families in germinating seeds. Fig. S2 Characterization of sweet1 mutants. Fig. S3 Expression profile of SWEET1. Fig. S4 SWEET1 levels under treatments and sweet1 phenotypes respond to different sugars. Fig. S5 Compromised Glc response in sweet1. Fig. S6 Expression of SnRK1‐related genes upon treatments in germinating seeds. Fig. S7 ABI5 unlikely contributing to Glc antagonizing ABA inhibition on seed germination. Fig. S8 TOR lacking contribution to Glc suppression of ABA inhibition on seed germination. Fig. S9 Downstream genes shared by ABA and KIN10. Please note: Wiley is not responsible for the content or functionality of any Supporting Information supplied by the authors. Any queries (other than missing material) should be directed to the New Phytologist Central Office. [file NPH-249-1816-s003.pdf]

New Phytologist Supporting Information

Article title: SWEET1-mediated glucose transport is crucial for energy availability in Arabidopsis

Authors: Xueyi Xue, Jiankun Li, Ya-Chi Yu and Li-Qing Chen

Article acceptance date: 24 October 2025

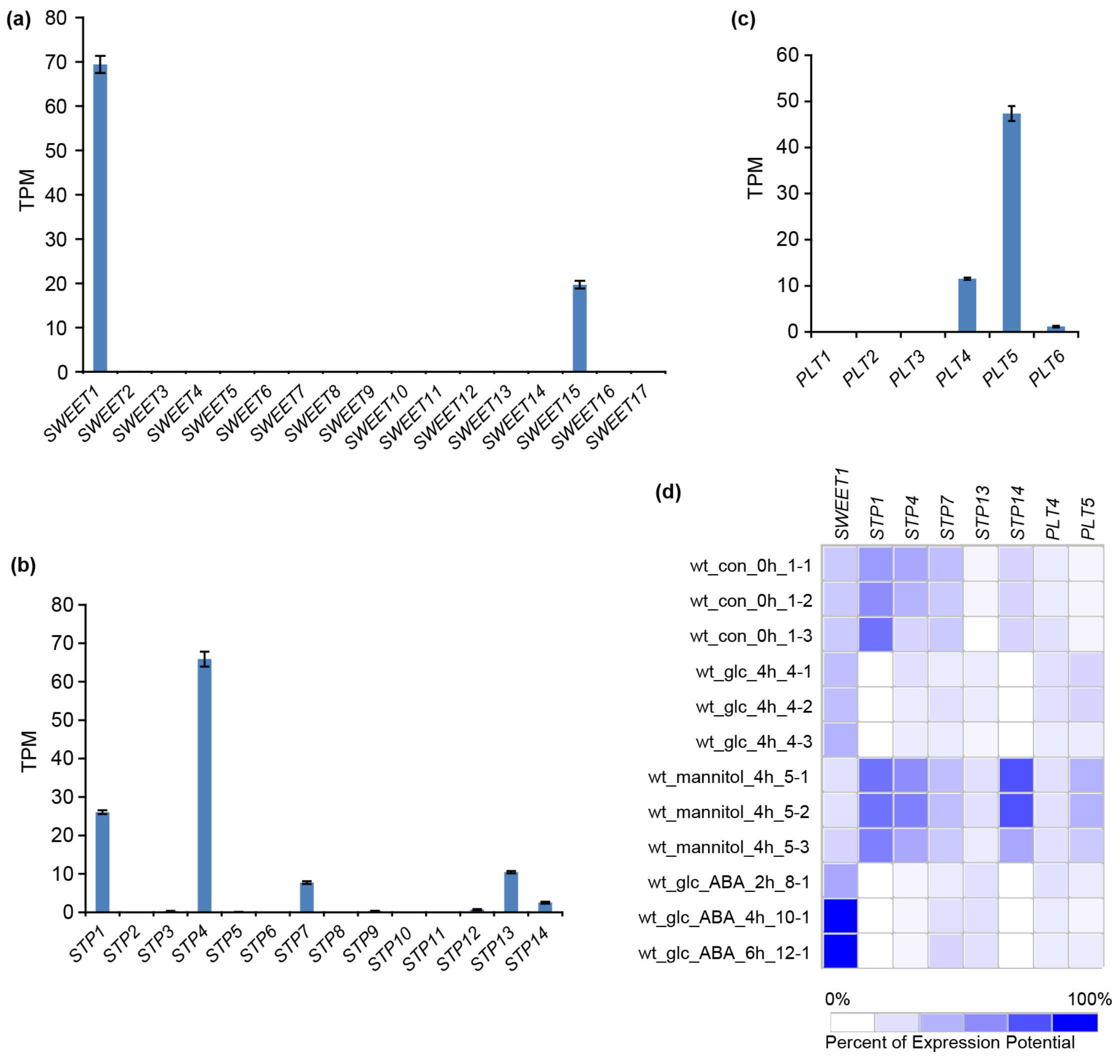

Fig. S1 Transcript levels of *SWEET*, *STP* and *PLT* families in germinating seeds. (a-c) Transcript levels of sugar transporter genes of *SWEETs* (a), *STPs* (b), and *PLTs* (c) at 30 HAI (Xue *et al.*, 2021). *SWEET1*, *SWEET15*, *STP1*, *STP4*, *STP7*, *STP13*, *PLT4*, and *PLT5* are relatively high. Values are means ± SD (n = 3).

(d) Expression of candidate Glc transporter genes upon Glc and ABA+Glc treatment in seedlings. These data were extracted from a study exploring the regulatory network of ABA and Glc in seedlings (Li *et al.*, 2006) through Genevestigator (Hruz *et al.*, 2008). *STP1*, *STP4*, *STP7*, and *STP14* were down-regulated by both Glc and ABA+Glc. *PLT4* and *PLT5* remained unchanged by ABA+Glc, while *SWEET1* was induced by ABA+Glc.

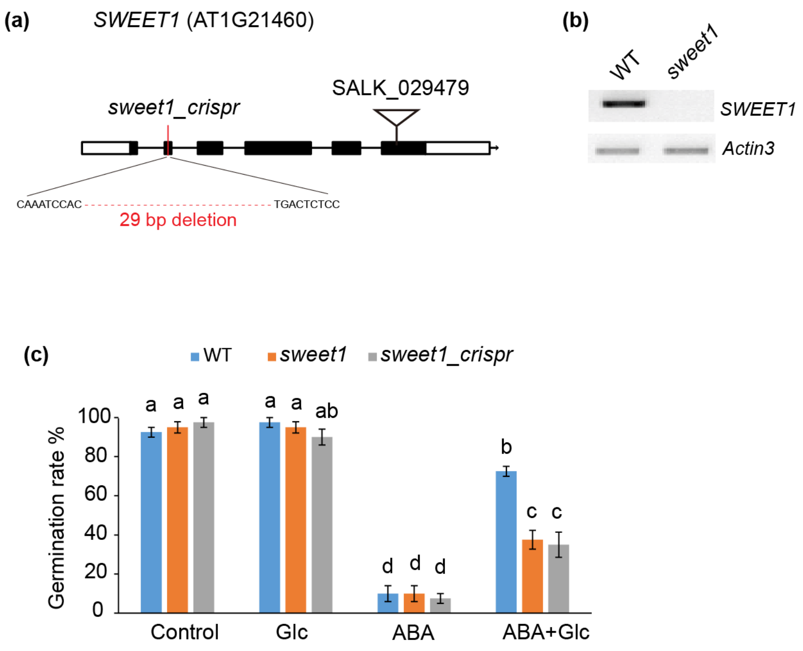

Fig. S2 Characterization of *sweet1* mutants.

(a) Schematic representation of *SWEET1* with the T-DNA insertion and CRISPR/Cas9 editing. The black boxes represent exons, and the white boxes represent UTRs. The triangle labels the T-DNA insertion site of allele SALK_029479. A 29 bp fragment was deleted from second exon in *sweet1_cripsr* allele.

(b) RT-PCR test for *SWEET1* transcript in *sweet1* mutant SALK_029479. There was no detectable *SWEET1* transcript in mutant seeds.

(c) Germination rate of WT and *sweet1* mutants under treatments at 72 HAI. Glc fails to promote germination of *sweet1* mutants in the presence of ABA. Values are means ± SE (n = 4). Different lowercase letters indicate significantly different means at $P <$ 0.05, as determined by a one-way ANOVA with Tukey test.

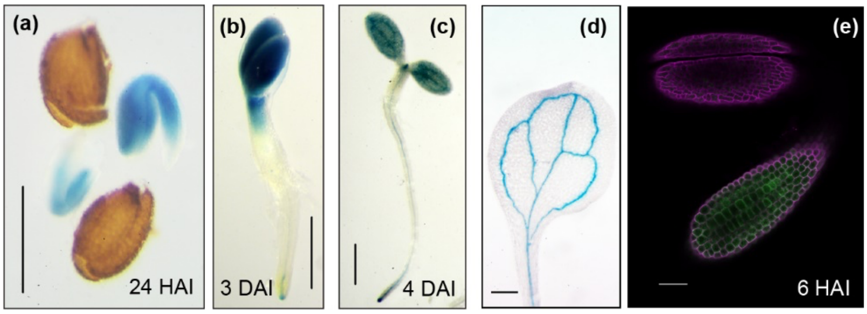

Fig. S3 Expression profile of *SWEET1*.

(a-c) SWEET1 accumulation pattern from germinating seeds to seedlings. GUS staining of *pSWEET1:gSWEET1-GUS* was observed in embryo, but not endosperm or testa at 24 HAI (a), seedlings (b,c) at indicated stages after imbibition. Scale bars = 0.5 mm.

(d) SWEET1 GUS staining in the first pair of true leaves from 3-week-old plants. Scale bar = 1 mm.

(e) SWEET1-YFP detected in cortex cells in hypocotyl at 6 HAI. Scale bar = 50 µm.

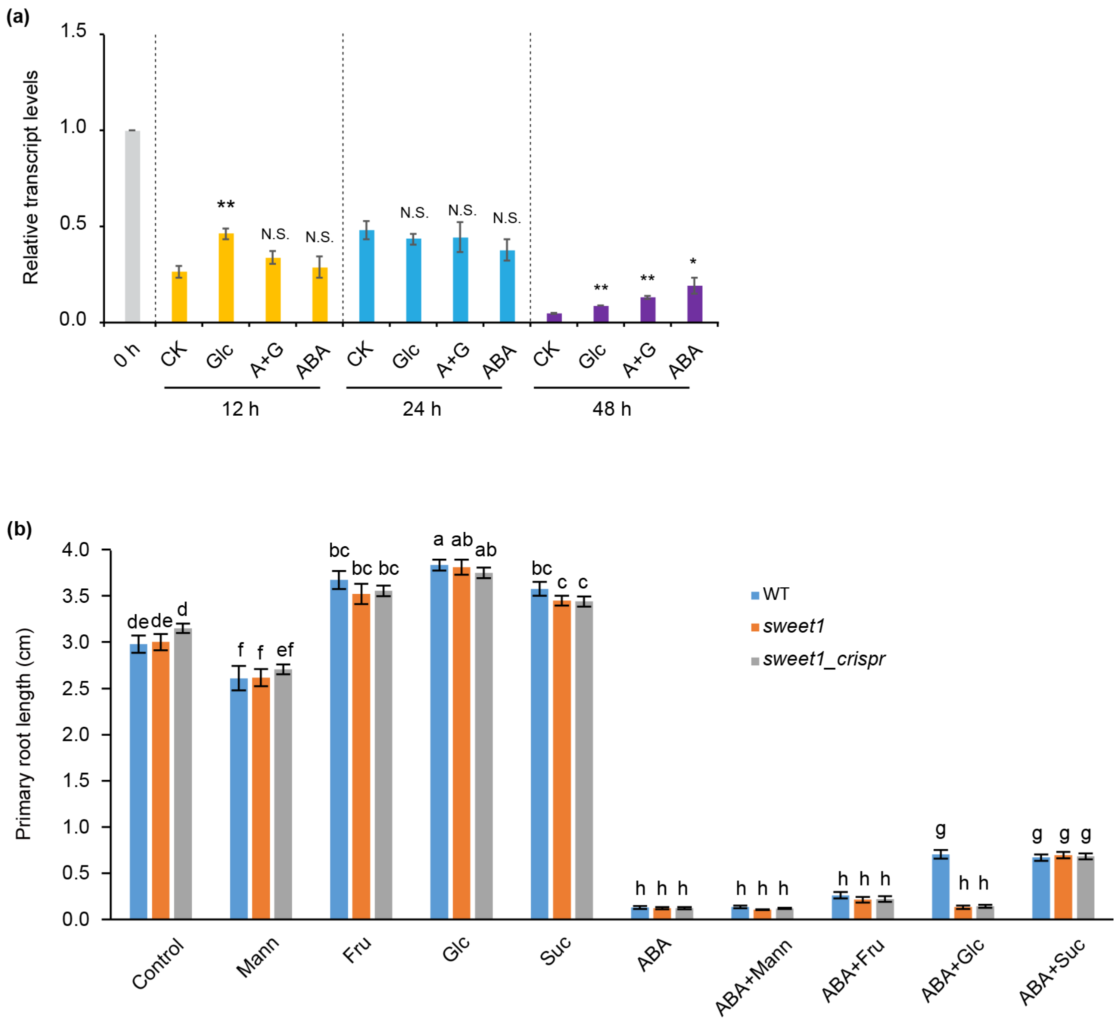

Fig. S4 *SWEET1* levels under treatments and *sweet1* phenotypes respond to different sugars.

(a) Transcript levels of *SWEET1* upon indicated treatments. After 3-day imbibition, Transcript levels were analyzed under treatments for 0, 12, 24 and 48 hours. Asterisks indicate significantly different compared with control (CK) values for each time point (Student's *t*-test, * $P < 0.05$, ** $P < 0.01$).

(b) Metabolizable sugars promote primary root growth under ABA treatment. After 3-day imbibition, seeds were sown on ½ MS medium with either 2 µM ABA or 2 µM ABA plus 60 mM sugar as indicated. The root length was measured at 10 DAI. Fru, Glc and Suc can promote root growth without ABA in both WT and *sweet1* mutants, but mannitol (Mann) did not. In the presence of 2 µM ABA, Suc can accelerate root growth in both WT and *sweet1* mutants compared to that on ABA only, while Glc promotes root growth in WT only. Different lowercase letters indicate significant different means at $P < 0.05$ by one-way ANOVA Tukey test.

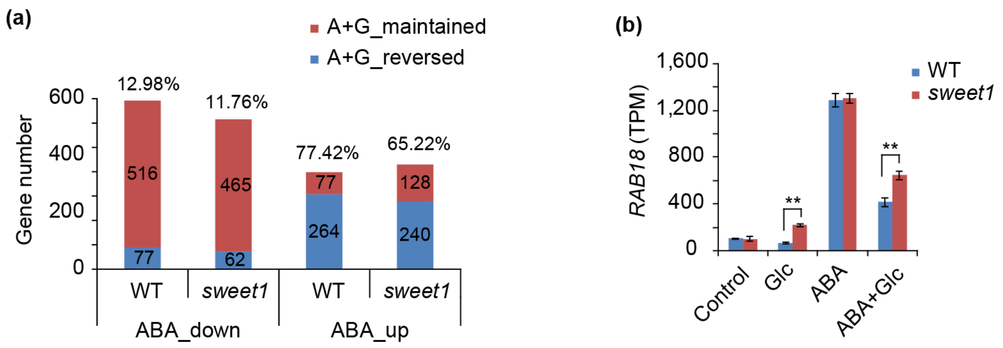

Fig. S5 Compromised Glc response in *sweet1*.

(a) The bar graph indicates the number of genes regulated by ABA in WT and *sweet1*. Genes with TPM > 20 under control condition and FC < 0.75 upon ABA treatment were classified as ABA down-regulated DEGs. Genes with TPM > 1.5 under control condition and FC > 2 upon ABA treatment were classified as ABA up-regulated DEGs. The numbers in the blue area indicate genes that showed the opposite change with the addition of Glc compared to ABA only. The number on top of each bar indicates the proportion of genes oppositely regulated by the addition of Glc.

(b) Expression levels of ABA-responsive gene *RAB18* under treatments. *RAB18* is highly induced by ABA and then reduced by the addition of Glc. Asterisks indicate significantly different compared with WT values (Student's *t*-test, ** $P < 0.01$).

(a)

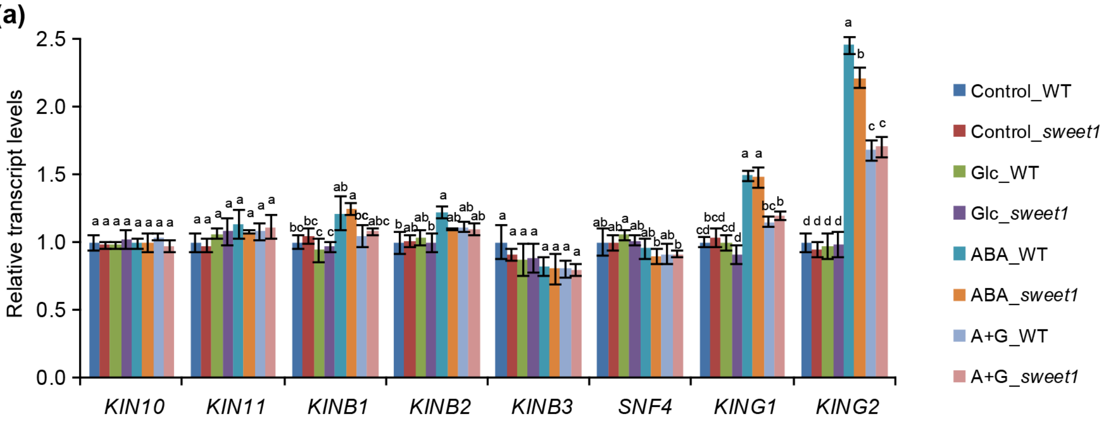

(b)

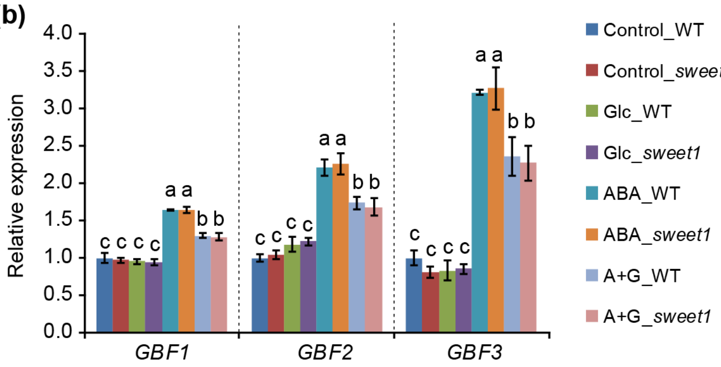

Fig. S6 Expression of SnRK1-related genes upon treatments in germinating seeds.

(a) Transcript levels of genes that encode subunits of the SnRK1 complex. The gamma subunit genes *KING1* and *KING2* were up-regulated by ABA and down-regulated by ABA+Glc relative to ABA. Genes encoding other subunits did not respond to the treatments.

(b) Transcript levels of *GBF* genes. *GBF1*, *2*, and *3* were induced by ABA and suppressed by the addition of Glc in both WT and *sweet1*. Different lowercase letters in (a,b) indicate significant different mean for each gene at $P < 0.05$ by one-way ANOVA Tukey test.

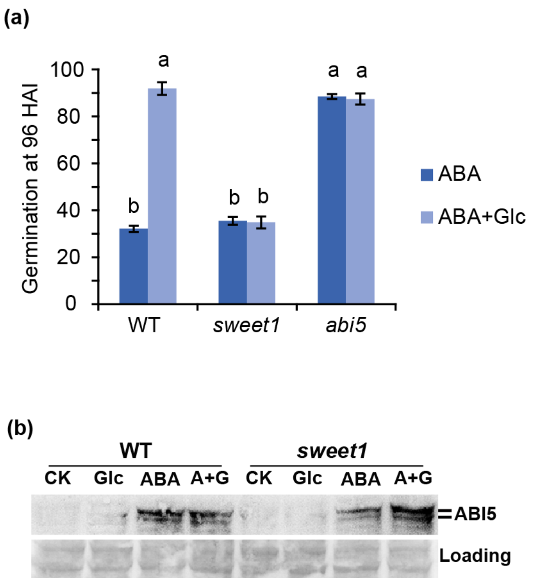

Fig. S7 ABI5 unlikely contributing to Glc antagonizing ABA inhibition on seed germination.

(a) Germination rate of WT, *sweet1*, and *abi5-8* under treatments at 96 HAI. The *abi5-8* is insensitive to ABA and ABA+Glc treatment. Values are means ± SE (n = 3). Different lowercase letters indicate significantly different means at $P < 0.05$ according to one-way ANOVA Tukey test.

(b) Immunoblotting shows ABI5 levels responding to treatments. ABI5 accumulation was up-regulated to a comparable level in both WT and *sweet1* mutants by ABA and ABA+Glc.

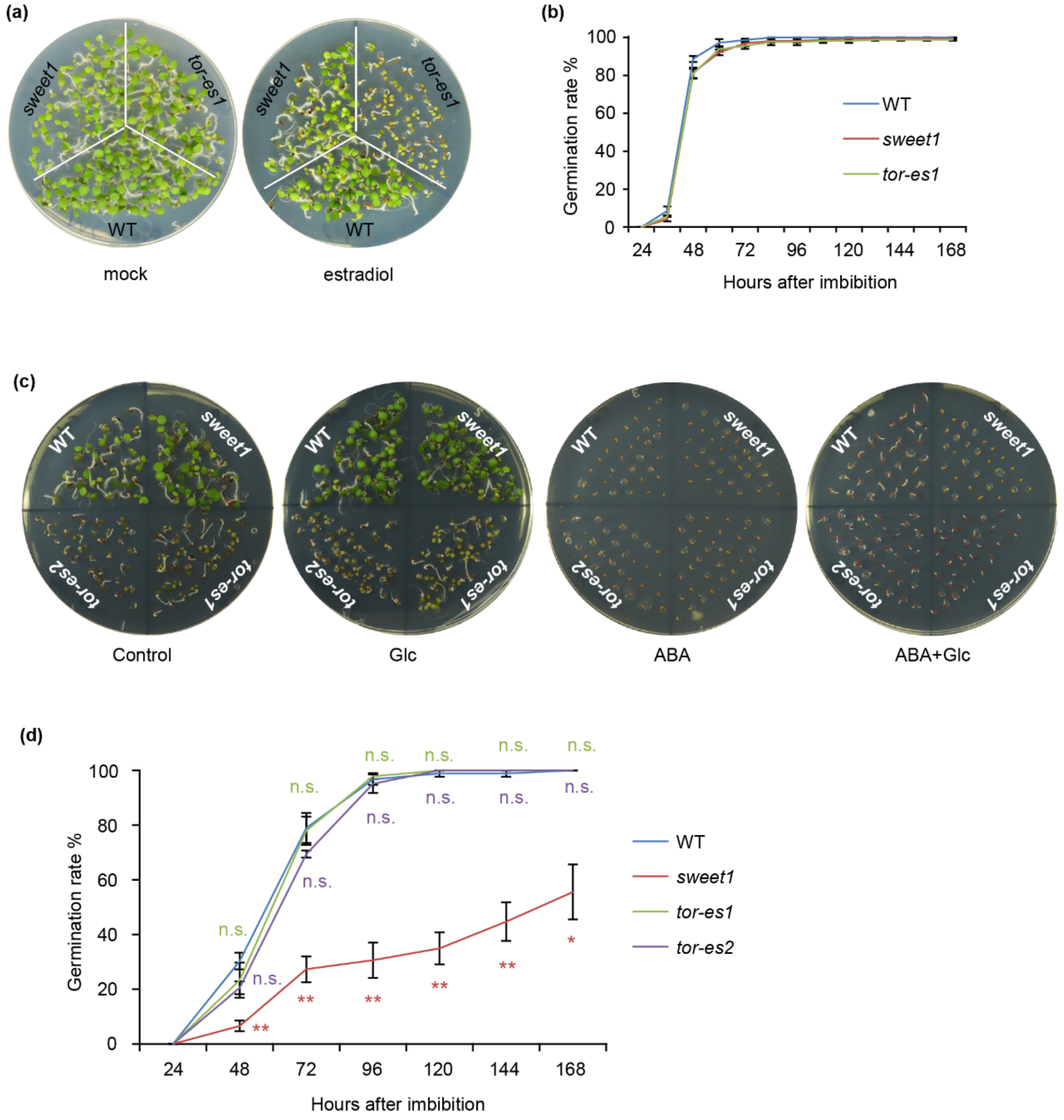

Fig. S8 TOR lacking contribution to Glc suppression of ABA inhibition on seed germination.

(a) Characterization of *tor-es1* phenotypes. Under 10 µM estradiol treatment, *tor-es1* showed stunted growth of cotyledons, true leaves, petioles, and roots as previously reported (Xiong & Sheen, 2012).

(b) Germination rate of WT, *sweet1*, and *tor-es1* under control conditions. WT, *sweet1* and *tor-es1* showed comparable germination rate on ½ MS medium supplemented with 10 µM estradiol. Values are means ± SE (n = 3).

(c) Phenotypes of two independent *tor-es* lines upon treatments for 7 days. Seeds of WT, *sweet1*, and two independent *tor-es* lines were grown on indicated medium.

(d) Germination rate of WT, *sweet1*, and two *tor-es* lines under ABA+Glc. WT, *tor-es1* and *tor-es2* showed comparable germination rate. Values are means ± SE (n = 3). Asterisks indicate a significant difference compared with WT values (Student's *t*-test, * $P < 0.05$, ** $P < 0.01$). n.s., no significant difference.

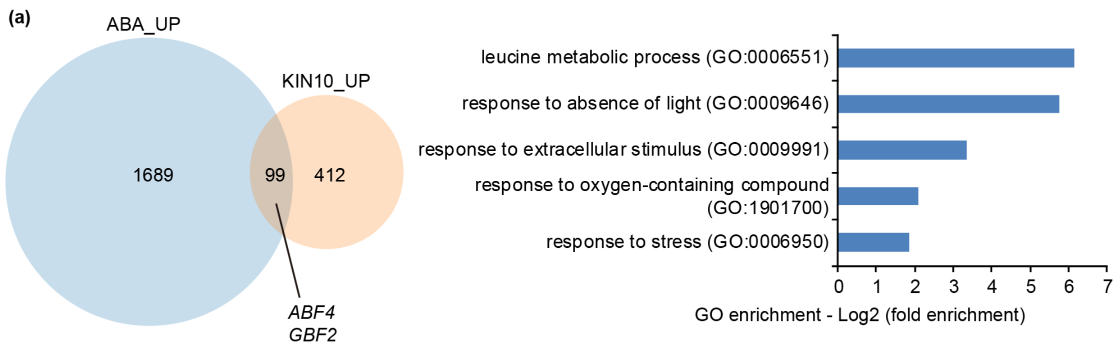

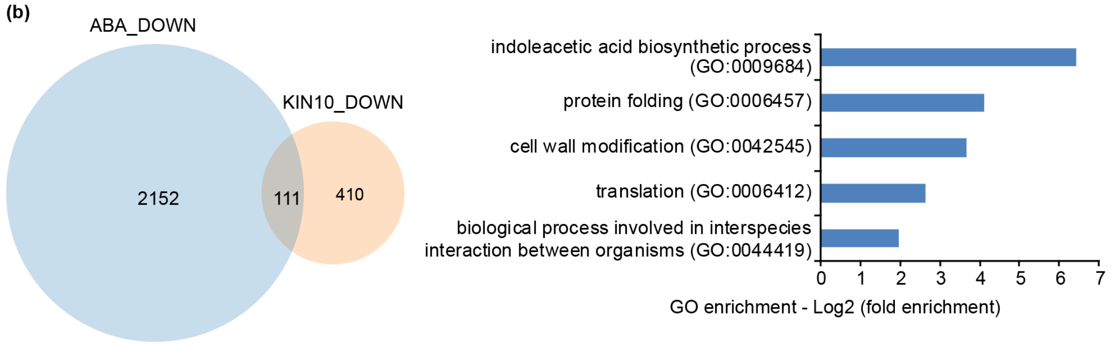

Fig. S9 Downstream genes shared by ABA and KIN10.

(a) Genes up-regulated by ABA and KIN10. Among 511 genes up-regulated by KIN10, 99 are also up-regulated by ABA in germinating seeds, including *ABF4* and *GBF2*. These overlapping genes play a role in the leucine catabolic process, response to the absence of light, extracellular stimulus, oxygen-containing compounds, and stress.

(b) Common genes down-regulated by ABA and KIN10. Of the 521 genes down-regulated by KIN10, 111 are also suppressed by ABA, including genes involved in indoleacetic acid biosynthesis, protein folding, cell wall modification, and translation. KIN10 response genes in (a,b) were downloaded from a previous study (Baena-Gonzalez *et al.*, 2007).

REFERENCES

Baena-Gonzalez E, Rolland F, Thevelein JM, Sheen J. 2007. A central integrator of transcription networks in plant stress and energy signalling. *Nature* **448**(7156): 938-942.

Hruz T, Laule O, Szabo G, Wessendorp F, Bleuler S, Oertle L, Widmayer P, Gruissem W, Zimmermann P. 2008. Genevestigator v3: a reference expression database for the meta-analysis of transcriptomes. *Adv Bioinformatics* **2008**: 420747.

Li Y, Lee KK, Walsh S, Smith C, Hadingham S, Sorefan K, Cawley G, Bevan MW. 2006. Establishing glucose- and ABA-regulated transcription networks in *Arabidopsis* by microarray analysis and promoter classification using a Relevance Vector Machine. *Genome Res* **16**(3): 414-427.

Xiong Y, Sheen J. 2012. Rapamycin and glucose-target of rapamycin (TOR) protein signaling in plants. *J Biol Chem* **287**(4): 2836-2842.

Xue X, Yu Y-C, Wu Y, Xue H, Chen L-Q. 2021. Locally restricted glucose availability in the embryonic hypocotyl determines seed germination under ABA treatment. *bioRxiv*: 2021.2004.2007.438879.
